# Spinal lesions: a comprehensive radiologic overview

**DOI:** 10.3389/fradi.2025.1577840

**Published:** 2025-06-27

**Authors:** Zahin Alam, Mohammed Usman Syed, Tausif Ahmed Siddiqui, Aditya Gunturi, Brij Reddy, Zarah Alam, Akm A. Rahman

**Affiliations:** ^1^ Department of Radiology, Hackensack Meridian School of Medicine, Nutley, NJ, United States; ^2^ Department of Imaging Sciences, University of Rochester Medical Center, Rochester, NY, United States; ^3^ Department of Biology, Stony Brook University, Stony Brook, NY, United States

**Keywords:** spinal lesions, MRI imaging, neoplastic and infectious pathology, spinal tumors, radiological assessment

## Abstract

Spinal lesions encompass a diverse range of pathologies, including primary and secondary tumors, infectious processes, vascular malformations, traumatic injuries, and degenerative conditions, each with distinct imaging characteristics crucial for accurate diagnosis and management. Imaging plays vital roles in assessing lesion morphology, anatomical localization, and neurological impact, guiding clinical decision-making and therapeutic planning. This review systematically explores spinal lesions based on their anatomical compartments, highlighting key radiological features and providing a comprehensive reference for radiologists.

## 1 Introduction

While primary spinal lesions are relatively rare, they remain a significant clinical concern due to their potential to cause severe morbidity, including chronic pain and dysfunction in motor and sensory function. These lesions can develop either within the spinal cord or from surrounding structures. One form of spinal lesions often manifests through the development of tumors. These tumors are typically categorized into primary tumors, which originate in the spine, and secondary tumors, which are metastatic in nature. Metastatic spinal tumors are much more common than primary tumors and often spread to the vertebral column through the bloodstream (1–3). Thus, it is imperative that radiological imaging is utilized for correct diagnosis, so patients receive prompt treatment to mitigate complications.

Various spinal lesions display unique imaging characteristics on CT and MRI, aiding in diagnosis and treatment planning (Table 1). Key features include anatomical location, invasion into adjacent structures, metastasis, and contrast enhancement (3). CT excels in visualizing bone involvement, highlighting both destructive patterns in high-grade malignancies and expansile growth in benign or low-grade tumors (4). MRI offers superior soft tissue contrast, making it ideal for detecting perineural spread and dural invasion (3, 4). These imaging findings highlight the importance of CT and MRI, which play vital roles in spinal lesion assessment.

**Table 1:** Imaging characteristics of spinal lesions by compartment.

Location	Lesion	Highlighted Imaging Characteristics
Intramedullary Region	Astrocytoma	Contrast enhancing eccentrically located tumors with irregular margins causing cord expansion.
		**T2WI:** Hyperintense
		**T1WI:** Varying contrast enhancement depending on tumor grade
	Ependymoma	Circumscribed, enhancing hemorrhagic masses often with cystic components and surrounding edema.
		“Cap sign” characterized by hemosiderin deposition at the cranial or caudal margins of the tumor indicating prior hemorrhage may be present.
		**T2WI:** Hyperintense
		**T1WI:** Isointense to slightly hypointense. Typically contrast enhancing
	Hemangioblastoma	Subpial enhancing nodule near the pial surface of the spinal cord often with flow voids, surrounding edema, and syrinx, a cystic cavity extending beyond the tumor margins contributing to cord expansion. Most often located in the cervical and thoracic regions.
		**T2WI:** Hyperintense
		**T1WI:** Strong, homogenous contrast enhancement
	Spinal Arteriovenous Malformation (AVM)	Well-defined highly vascular, serpentine flow voids within an enlarged heterogeneously hyperintense spinal cord. Magnetic resonance angiography (MRA) can define the key vascular components, including enlarged feeding arteries, the central nidus, and enlarged draining veins. Definitive diagnosis is by digital subtraction angiography (DSA)
		**T2WI:** Hyperintense, flow voids
		**T1WI:** Flow voids, dilated vessels
	Traumatic Spinal Cord Injury	Hyperintense, multi-segment cord expansion indicates edema while low intensity foci within edematous region indicate hemorrhage suggesting worse prognosis.
		**T2WI:** Hyperintense edema possibly surrounding low intensity foci of hemorrhage
		**T1WI:** Isointense cord expansion
	Syringomyelia	Enlarged spinal cord due to the dilated, beaded, or sacculated cystic cavity within the spinal parenchyma or central canal. Reversible pre-syrinx state with T2-hyperintense edema without cavitation may precede syrinx formation. Cerebellar tonsillar ectopia may be seen in Chiari malformation type I (CM1).
		**T2WI:** Hyperintense
		**T1WI:** Hypointense
	Spinal Cord Infarction	T2 hyperintensities commonly involving anterior two-thirds of the spinal cord due to anterior spinal artery syndrome (ASAS). Extensive involvement across multiple levels may present as “owl eye” sign.
		**T2WI:** Hyperintense
		**T1WI:** Subacute contrast enhancement
		**DWI:** Early ischemia identified by restricted diffusion
		**STIR:** Identification of bone infarcts
	Transverse Myelitis	Long stretch of T2 hyperintensity involving >3 vertebral segments occupying more than two-thirds of the spinal cord's cross-sectional area. Presents with variable enhancement, cord expansion, and no diffusion restriction.
		**T2WI:** Hyperintense
		**T1WI:** Iso-hypointense with variable contrast enhancement
	Demyelinating Disease	Scattered, wedge-shaped, T2 hyperintense lesions larger than 3 mm, located peripherally and extending less than three vertebral levels with contrast enhancement of active lesions.
		**T2WI:** Hyperintense
		**T1WI:** Hypointense. Active lesions enhance with contrast
Intradural Extramedullary Space	Meningioma	Often located laterally to the spinal cord, with the thoracic spine being the most common site (80%). The “ginkgo leaf sign” on T1WI with contrast representing the displaced spinal cord aids in differentiating meningioma from schwannoma.
		**T2WI:** Hyperintense
		**T1WI:** Hypointense with possible contrast enhancement
	Schwannoma	Oval or round well-circumscribed, dumbbell-shaped transforaminal masses, primarily affecting the cervical and lumbar regions, where they originate from dorsal sensory roots.
		**T2WI:** Hyperintense, heterogeneous
		**T1WI:** Isointense or hypointense
	Neurofibroma	Occur along the dorsal sensory roots leading to radiculopathy or myelopathy and potential remodeling of adjacent bones, widening of the neural exit foramen, and scalloping of the vertebrae
		May display nonspecific “target sign” on T2WI as central hypointensity with hyperintense rim.
		**T2WI:** Isointense to hyperintense
		**T1WI:** Isointense
Epidural Space	Spinal Epidural Abscess	Localized focus of high T2 hyperintensity and rim enhancing T1 hypointensity. Compression or inflammation of adjacent structures, such as the spinal cord, nerve roots, vertebral bodies, or discs, may be present.
		**T2WI:** Hyperintense
		**T1WI:** Hypointense with peripheral contrast enhancement
	Spinal Epidural Hematoma	Loss of normal MRI epidural fat signal with a smooth contour adjacent to the spinal cord. Fluctuating MRI characteristics over hyperacute, acute, early-late subacute, and chronic phases.
		Hyperacute phase,
		**T2WI:** Hyperintense with possible hypointense rim
		**T1WI:** Isointense
		Acute phase,
		**T2WI:** Hypointense
		**T1WI:** Hypo-isointense
		Early subacute phase,
		**T2WI:** Hypointense
		**T1WI:** Hyperintense
		Late subacute phase,
		**T2WI:** Hyperintense
		**T1WI:** Hyperintense
		Chronic phase,
		**T2WI:** Hypointense
		**T1WI:** Hypointense
	Spinal Epidural Tumor	Both primary tumors and metastases may present in the epidural space. Most epidural spinal metastases are located in the thoracic spine and may lead to spinal cord compression. Different tumors display a range of intensities and enhancement patterns on T1 and T2 weighted MRI depending on the tumor content.
		**T2WI:** Variable intensity
		**T1WI:** Variable intensity, with potential contrast enhancement useful to delineate extent of lesion
		**CT:** Characterize bone and vertebral involvement
Intervertebral Disc	Intervertebral Disc Degeneration	Loss of MR signal intensity at site of degenerative changes. The most commonly affected intervertebral spaces are C5/6, T6/7, and L4/5.
		**T2WI:** Hypointense changes within disc
		**T1WI:** Bone marrow changes surrounding disc with variable intensities
	Disc Herniation	Displaced intervertebral disc material includes four types of lesions: protrusion, extrusion, migration, and sequestration. Protrusions are confined to the disc level with an intact annulus, while extrusions show annular tears with nuclear material extending past endplates. MRI is used to delineate extent of herniation. CT is used to identify bone involvement and fractures.
	Discitis Osteomyelitis	Narrowing of the intervertebral disc space and destruction of adjacent vertebral endplates indicative of infectious or inflammatory involvement. Adjacent vertebral bodies may appear T2 hyperintense due to bone marrow edema, and T1 contrast-enhanced MRI can further delineate the extent of infection or abscess formation.
		**T2WI:** Hyperintensity in affected disc
		**T1WI:** Hypointense in affected disc and adjacent vertebral bodies with contrast enhancement
		**CT:** Cortical bone destruction
Vertebral Region	Vertebral Hemangioma (VH)	Typical VH are slow growing without extraosseous extension with “corduroy cloth”, “polka-dot”, or “salt and pepper” signs.
		**T2WI:** Hyperintense
		**T1WI:** Hyperintense with variable contrast enhancement
		Atypical VH are also more likely to be aggressive without “corduroy” or “polka-dot” signs
		**T2WI:** Hyperintense
		**T1WI:** Iso-hypointense
		Aggressive VH resemble atypical VH and may extend beyond vertebral body causing bone destruction
		**T2WI:** Hyperintense
		**T1WI:** Hypointense
		**CT:** Honeycomb pattern within vertebral body
	Osteoid Osteoma (OO) & Osteoblastoma	OO commonly presents in the lumbar region on CT with a characteristic central, lucent nidus <1.5–2 cm of variable contrast enhancement that may be absent on MRI.
		Osteoblastoma also often presents in the lumbar region and are >4 cm with a thin marginal bone shell, central intralesional mineralization, cortical destruction, expansile bone remodeling, and reactive sclerosis peripherally on CT. MRI may overestimate the lesion extent due to inflammation.
		**T2WI:** Hyper-isointense
		**T1WI:** Hypo-isointense
	Osteosarcoma	Mixed osteoblastic/osteosclerotic and osteolytic lesions with components of variable ossificationtypically affecting posterior spinal elements on CT.
		Highly mineralized tumors may present as “ivory vertebra”. Intensities on MRI may be nonspecific.
		**T2WI:** Hypointense for mineralized and hyperintense non-mineralized tumors
		**T1WI:** Hypointense for mineralized tumors
		**CT:** Used to characterize mineralization pattern and cortical destruction
	Vertebral Metastases	Foci of elevated radiotracer uptake on bone scintigraphy. FDG PET CT is highly sensitive and specific for detecting metastases.
		**T2WI:** Hyperintense osseous metastases with bright rim (Halo sign).
		**T1WI:** Hypointense bone marrow lesions. Fat suppression increases intensity of metastases.
		**CT:** Used to delineate cortical bone destruction
	Multiple Myeloma	Focal well-circumscribed lytic bone lesions without reactive sclerosis or diffuse osteolysispossibly involving vertebral bodies with extension to pedicles and disc space. CT is preferred for fracture risk assessment. On MR, normal and “salt and pepper” appearances indicate stage I disease, while diffuse infiltration, focal lesions, and combined focal/diffuse infiltration indicate higher stage disease.
		**T2WI:** Typically hypointense, but hyperintense with fat suppression
		**T1WI:** Typically hypointense, and contrast enhancing
	Spinal Lymphoma	Lytic, mixed, or rarely sclerotic bone lesions with cortical thickening, periosteal reaction, or pathologic fracture present on CT. Commonly, anterior vertebral body involvement with preserved disc space occurs. FDG PET/CT may show focal or multifocal radiotracer uptake with a non-focal/diffuse background.
		**T2WI:** Hyperintense with fat suppression
		**T1WI:** Hypointense with variable contrast enhancement
	Vertebral Compression Fracture	The mid-thoracic (T7–T8) and thoracolumbar (T12–L1) regions are most often affected. End plate deformities, altered/non-uniform vertebral size/shape, or loss of vertebral height > 20% due to wedge, biconcave, or crush deformities may be present on radiographs, CT, and MRI.
Prevertebral Space	Prevertebral Space Neoplasm	May involve primary neoplasms or metastases with differing imaging characteristics. Primary neoplasms such as leukemia, lymphoma, and multiple myeloma often affect bones, while common benign lesions such as lipoma are T1 hyperintense and saturated with fat suppression. Metastatic lesions may result in mass effect, cord compression, and present on MRI as:
		**T2WI:** Hyperintense
		**T1WI:** Hypointense with contrast enhancement
	Retropharyngeal Abscess	Increased prevertebral space thickness on radiographs, but CT or MRI is preferred.
		**T2WI:** Hyperintense
		**T1WI:** Hypointense with rim contrast enhancement
Incidental Lesions	Congenital Vertebral	Block vertebrae present with absence of the normal intervertebral disc and a continuous fusion of the vertebral bodies on MRI.
		Chondrification or ossification failure may present as hemivertebrae or “butterfly vertebra” in cases of failed sagittal fusion of the of a vertebral body.

**Figure 1:**
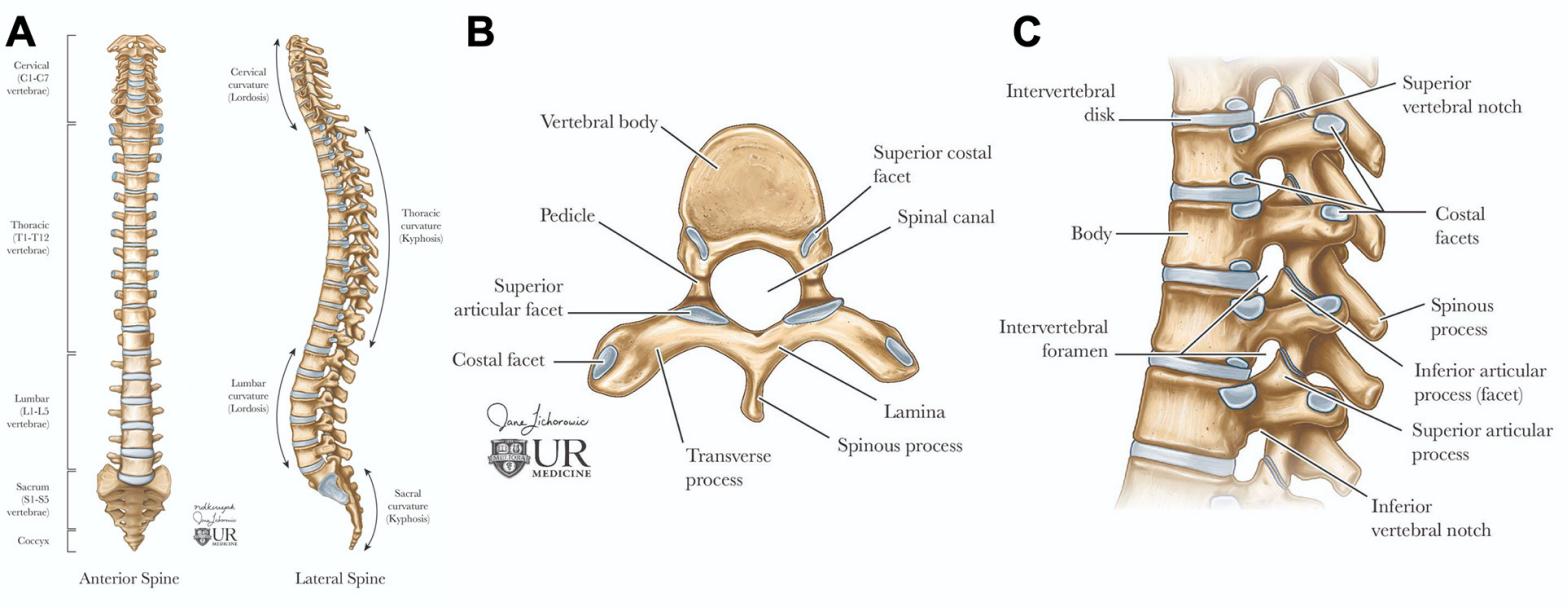
Spinal anatomy. The adult vertebral column **(A)** consists of 24 vertebrae, plus the sacrum and coccyx. It is divided into cervical (C1–C7), thoracic (T1–T12), and lumbar (L1–L5) regions. Axial view of the vertebral body **(B)** illustrates the arch formed by pedicles and laminae. Sagittal view of the vertebral column **(C)** shows the alignment of the vertebral body and intervertebral foramina for spinal nerve exit, the vertebral foramen for spinal cord passage, and intervertebral discs connecting adjacent bodies.

The anatomy of the spine is complex and vital, consisting of 33 vertebrae categorized into five regions: cervical, thoracic, lumbar, sacral, and coccygeal. Its double-S shape, with lordotic curves in the cervical and lumbar regions and kyphotic curves in the thoracic and sacral regions, enhances strength, flexibility, and shock absorption (Figure 1A). This structure protects the spinal cord, supports weight transfer from the upper body to the pelvis, and provides muscle attachment sites (5). Cervical vertebrae (C1–C7) support the head with a wide range of motion; C1 (atlas) allows nodding, while C2 (axis) enables rotation. Thoracic vertebrae (T1–T12) articulate with ribs, and lumbar vertebrae (L1–L5) are larger for weight-bearing and movement. The sacrum connects to the pelvis and contains sacral nerves, while the coccyx serves as a site for pelvic floor muscle attachment (5).

Each vertebra features a cylindrical body connected by intervertebral discs, which have a gelatinous core (nucleus pulposus) and a tough outer layer (annulus fibrosus), contributing to about 25% of the spine's length (5, 6). The vertebral arch, formed by pedicles and laminae, encases the spinal cord and includes processes for muscle and ligament attachment, facilitating movement (Figure 1B) (5, 6). Muscles and spinal nerves are also integral to the spine's anatomy, contributing to its stability, functionality, and mobility. Intrinsic back muscles, innervated by the dorsal rami of spinal nerves, enable spinal movement, alongside muscles of the neck, shoulders, chest wall, diaphragm, abdomen, gluteal region, and pelvic floor. The spinal nerves include 31 pairs—8 cervical, 12 thoracic, 5 lumbar, 5 sacral, and 1 coccygeal—carrying autonomic, motor, and, through dermatomes, sensory signals (5, 6).

The prevertebral and paravertebral spaces, enclosed by deep cervical fascia, form the peri-vertebral space (7). The prevertebral space, defined by the vertebral bodies, contains prevertebral muscles, the brachial plexus, phrenic nerve, and vertebral vessels, while the paravertebral space, bordered by the vertebrae, houses paraspinal muscles (7). A solid understanding of spinal anatomy is crucial for the accurate radiologic diagnosis and management of spinal lesions. Each imaging modality, such as MRI and CT, offers unique advantages depending on the anatomical area of interest and the clinical question.

The objective of this article is to provide an exploration of a variety of spinal lesions, with a particular focus on their imaging characteristics and anatomical impacts to offer a concise and practical reference for radiologists, enhancing their understanding of the complex presentations of spinal lesions. The article begins by examining lesions originating within the spinal cord itself, then systematically progresses outward to cover lesions affecting various sections of the spine, including the vertebrae and adjacent structures. It further explores incidental lesions that may arise in neighboring areas, providing a comprehensive overview of the diverse spectrum of spinal pathology. By highlighting the imaging features associated with different spinal lesions, this article seeks to aid in the accurate diagnosis and assessment of these conditions.

## 2 Lesions involving the spinal cord

### 2.1 Spinal intramedullary tumors

Intramedullary tumors account for 4%–10% of all central nervous system (CNS) tumors, with approximately 80% of these being gliomas, predominantly astrocytoma and ependymomas (8, 9). Non-glial neoplasms include rare entities such as spinal hemangioblastoma, paraganglioma, intramedullary metastasis, and primary spinal lymphoma. Additionally, benign masses like spinal epidermoid cysts and lipomas may also be present within the spinal cord. Clinically, patients often experience a diffuse burning pain that worsens at night, along with symptoms such as paresthesia, motor weakness, clumsiness (especially in children), and autonomic dysfunction (10). MRI remains the preferred imaging modality for diagnosing intramedullary tumors due to its superior ability to visualize spinal cord lesions. The mainstay of treatment is surgical resection, aiming to preserve neurological function and to remove as much of the tumor as safely possible (8, 11).

#### 2.1.1 Astrocytoma

**Figure 2:**
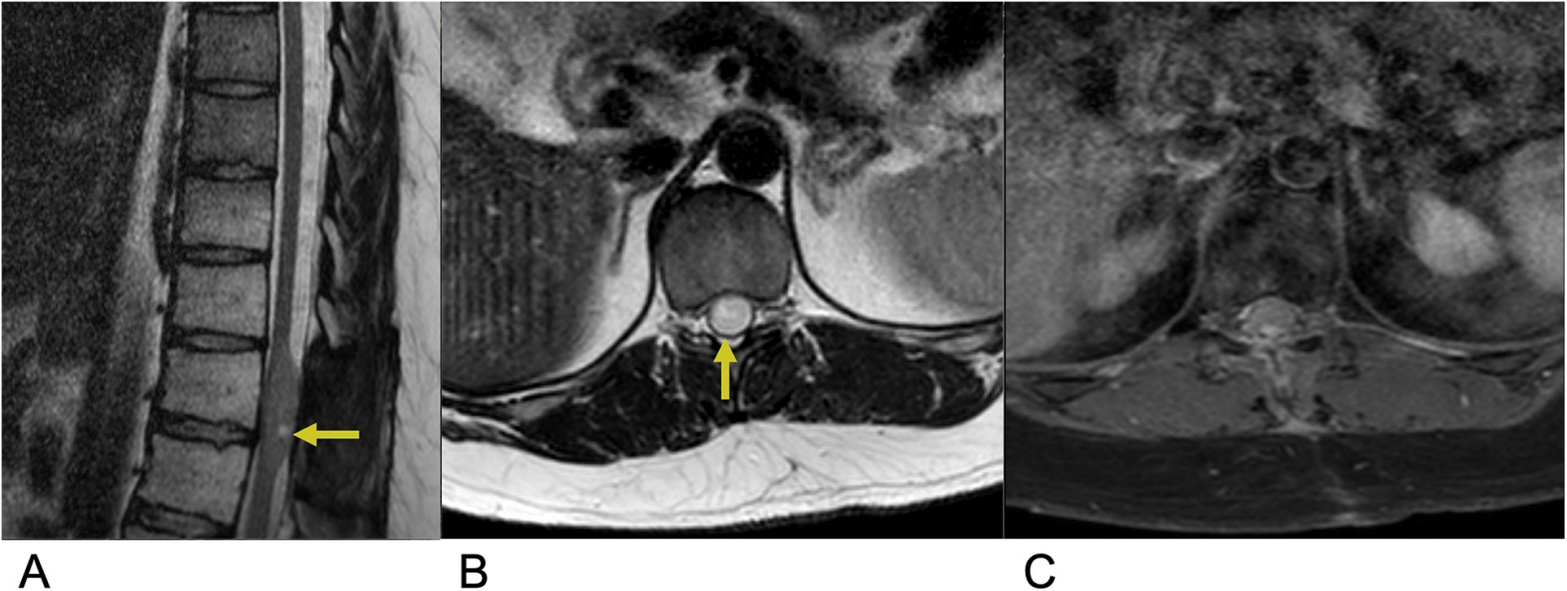
Astrocytoma. Sagittal T2 **(A)**, axial T2 **(B)**, axial T1 FS + C **(C)** MRI of the thoracic spine demonstrating an intramedullary expansile T2 hyperintense mass (yellow arrows) in the thoracic cord at the T10–T11 level without post-contrast enhancement representing biopsy-proven Grade 1 Pilocytic Astrocytoma.

Astrocytoma is the abnormal proliferation of astrocytes, star-shaped glial cells that conduct various functions in the central nervous system, such as regulating the blood brain barrier and maintaining homeostasis (12). Primary spinal astrocytoma accounts for a third of spinal cord gliomas (13, 14). Among spinal cord gliomas, spinal astrocytoma is the most common spinal cord tumor found in the intradural intramedullary compartment and are much more common in the pediatric age group (10, 15). The vast majority, around 75%–80%, are low grade pilocytic astrocytoma while the rest, 20%–25%, are high grade anaplastic astrocytoma (16). There is a genetic association between neurofibromatosis type I and astrocytoma, predominantly affecting males (10, 17). Presentation includes back pain, motor weakness, sensory deficits, and sometimes bowel or bladder dysfunction, depending on the tumor's location and extent of spinal cord compression. Due to the infiltrative nature of astrocytoma, achieving total resection is often challenging (17, 18). As a result, the primary goal of treatment focuses on preserving neurological function and preventing further deterioration. A key imaging characteristic for astrocytoma is a predominantly enhancing intramedullary lesion, typically due to disruption of the blood-brain barrier. Another hallmark is cord expansion, where the spinal cord appears widened due to the tumor's presence. The tumor typically presents with ill-defined margins, making it challenging to distinguish the tumor from normal spinal cord tissue. Additionally, the lesion is often eccentric in location, meaning it is off-center within the spinal cord rather than symmetrically placed. On MRI, astrocytoma generally appears hyperintense on T2-weighted images due to the high-water content and can show varying degrees of contrast enhancement on T1-weighted post-contrast images, depending on the tumor grade (Figure 2) (19).

#### 2.1.2 Ependymoma

**Figure 3:**
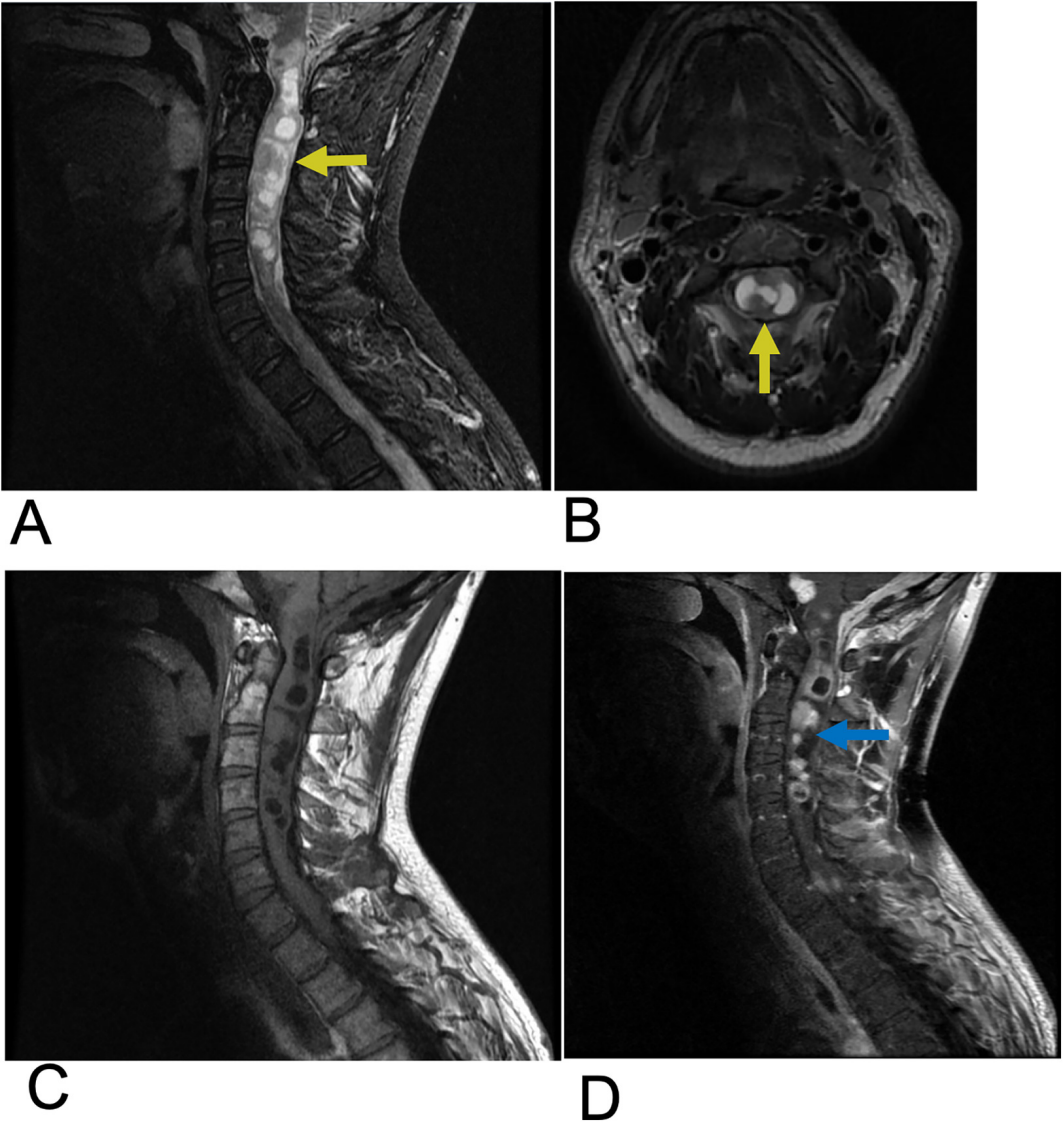
Ependymoma. Sagittal T2 **(A)**, Axial T2 **(B)**, Sagittal T1 FLAIR **(C)**, and sagittal T1 FLAIR FAT + C **(D)** MRI of the cervical spine demonstrating intramedullary expansile multiloculated T2 hyperintense cystic lesions (yellow arrows) in the cervical cord spanning from C2–C5 with edema at the inferior aspect of the lesions. There are associated enhancing tumoral nodules in the spinal cord representing spinal ependymoma (blue arrow). No evidence of hemorrhage.

Ependymoma are abnormal neoplasms that arises from the ependymal lining of the spinal cord central canal (20). Among adults, ependymoma are the most common intramedullary spinal cord tumors, consisting of 60% of all glial spinal cord tumors (20, 21). Males are slightly more affected than females, along with a higher incidence in white individuals compared to black individuals (20, 22). 50%–60% of adult ependymomas are located in the spine, with the cervical and lumbar sites, especially the filum terminale being most affected (20). Ependymoma has a similar presentation to other intramedullary spinal cord tumors with common symptoms including back pain, sensory loss, and paresthesia (23). Symptoms are variable depending on cervical vs. lumbar tumor location. Complete surgical resection is the first line of treatment and is often curative (23). Three subtypes of ependymoma are classified based on microscopy. Grade I lesions including Myxopapillary ependymoma and subependymoma are most benign. “Classic” ependymoma are Grade II and the most common type of ependymoma. Grade III lesions are anaplastic ependymoma and exhibit the most malignant and aggressive behavior (23, 24). Anaplastic ependymoma are the rarest form, and complete resection is challenging due to infiltrative behavior resulting in more frequent recurrence. MRI is the preferred imaging modality for ependymoma. These lesions are often characterized by imaging as circumscribed, enhancing hemorrhagic masses within the spinal cord frequently surrounded by edema. These tumors are commonly associated with cystic components. On MRI, ependymoma typically appear isointense or slightly hypointense to the spinal cord on T1-weighted images and hyperintense on T2-weighted images relative to the normal cord tissue. A distinguishing feature, known as the “cap sign”, may be present, characterized by hemosiderin deposition at the cranial or caudal margins of the tumor, indicating prior hemorrhage. Most spinal ependymomas exhibit enhancement following contrast administration, aiding in their identification and differentiation from other spinal cord lesions (Figure 3) (25, 26).

#### 2.1.3 Hemangioblastoma

**Figure 4:**
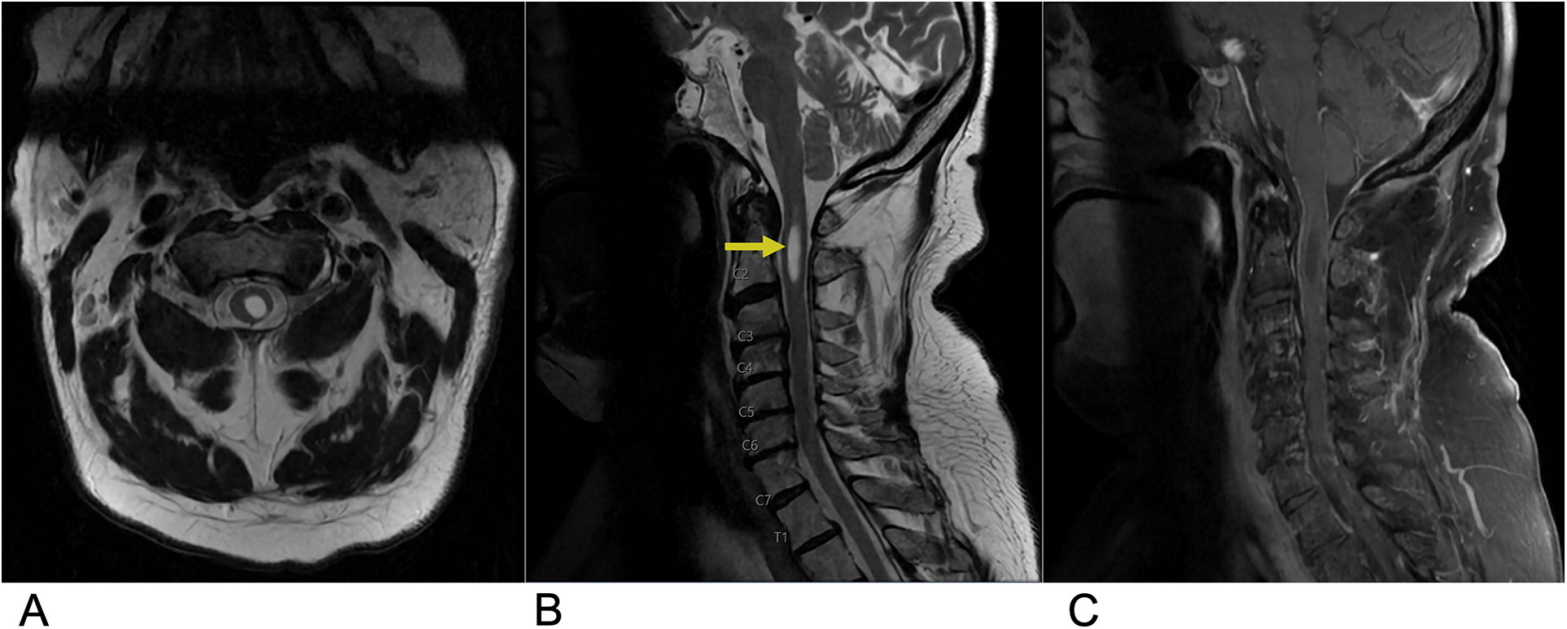
Hemangioblastoma. Axial T2 **(A)**, sagittal T2 **(B)**, & sagittal T1 FAT + C **(C)** MRI depicting a T2 hyperintense lesion (yellow arrow) at the C2 level representing a hemangioblastoma in this patient with a known history of Von Hippel Lindau Syndrome.

Hemangioblastoma are a rare benign, capillary-rich neoplasm of the cerebellum and spinal cord (27, 28). Spinal hemangioblastomas account for 2%–6% of all spinal cord tumors (29). These lesions are commonly seen in two-thirds of cases as a sporadic occurrence but can also be associated with von Hippel-Lindau (VHL) syndrome in approximately one-third of cases (29). VHL syndrome is a rare, inherited disorder that is characterized by multiple lesions that develop throughout the central nervous system and other organs such as the eyes, pancreas, and kidneys. Despite their low-grade nature, these lesions often result in significant neurological damage and high morbidity due to their sporadic distribution within the spinal cord and the mass effect they exert on surrounding neural structures (30, 31). Microsurgical resection of these tumors is the first-line treatment, followed by stereotactic radiosurgery for those with recurrent, incomplete, or residual lesions (27, 32). MR is the most effective imaging modality for evaluation of spinal hemangioblastoma (33). On T1-weighted MRI with contrast, hemangioblastoma typically present as brightly enhancing lesions due to their rich vascularity, often appearing as a subpial enhancing nodule near the pial surface of the spinal cord. The strong, homogeneous enhancement seen with gadolinium administration highlights the tumor's vascular nature. On T2-weighted imaging, hemangioblastoma are frequently associated with surrounding edema and a syrinx, a cystic cavity within the spinal cord that often extends beyond the tumor margins, contributing to cord expansion and neurological symptoms (27, 34). Additional imaging features include flow voids, which indicate high blood flow within the tumor, and these lesions are most commonly located in the cervical and thoracic regions (Figure 4) (34, 35).

#### 2.1.4 Spinal arteriovenous malformation (AVM)

**Figure 5:**
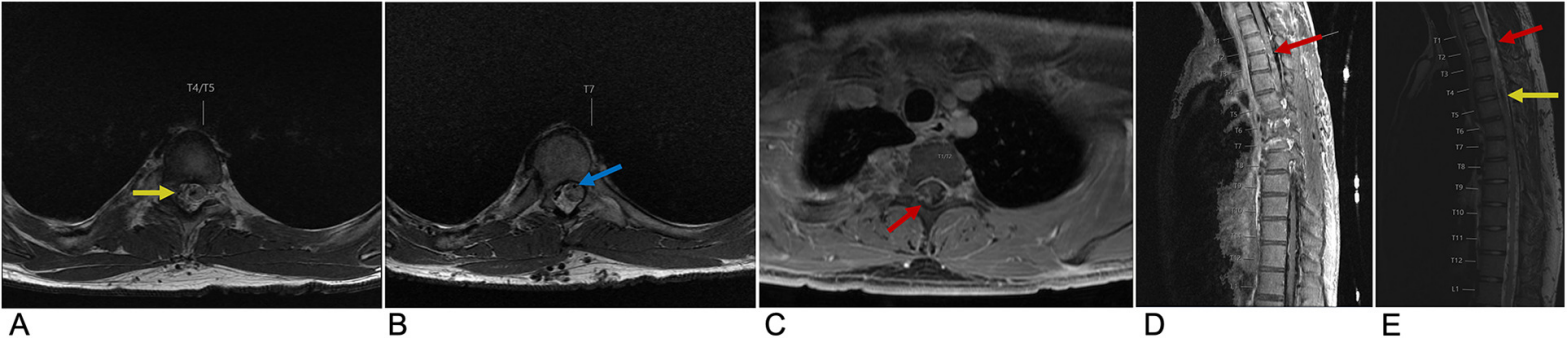
Spinal AVM. Axial T2 at the T4 level **(A)**, Axial T2 at the T7 level **(B)**, axial T1 + C at the T1/T2 level **(C)**, sagittal T2 **(D)**, and sagittal T1 FLAIR FAT + C **(E)** MRI of the thoracic spine demonstrating multifocal enhancement with associated expansion of the spinal cord (red arrow) involving the upper thoracic spine with prominent sub-adjacent flow voids (yellow arrow) within the right intradural and extradural space and dilation of the anterior spinal artery (blue arrow) representing spinal AVM.

Spinal AVMs are rare, abnormally developed spinal blood vessels that lead to vessel engorgement and thus are associated with an increased risk for subarachnoid hemorrhage and morbidity (36). These lesions are characterized by a nidus of abnormal blood vessels or fistula between these arteries and veins (37). Spinal AVMs are usually supplied by the anterior and/or posterior spinal arteries, with the nidus draining into the coronal venous plexus on the cord surface, which then drains anterograde into the extradural space (38). These particular spinal vessel defects shunt arteriole blood to the venous system without capillary access and resistance, culminating in venous hypertension, precipitating neurological deficiencies secondary to mass effect and blood flow disruption (36, 37). Spinal AVMs account for 3%–4% of all intradural spinal cord mass lesions and mainly affect patients in the 40–65-year age range (37). The definitive diagnosis for spinal AVMs is made with Digital Subtraction Angiography (DSA). However, DSA is a risky procedure with several complications. Thus, MRI is the initial non-invasive imaging modality of choice (36). These complex vascular lesions are characterized by multiple well-defined serpentine flow voids within the spinal cord substance and typically present with a large, heterogeneously hyperintense spinal cord containing blood products and multiple flow voids due to their highly vascular nature. Magnetic resonance angiography (MRA) supplements MRI, and can define the key vascular components, including enlarged feeding arteries, the central nidus, and enlarged draining veins (Figure 5) (36, 39).

### 2.2 Spinal intramedullary lesions

#### 2.2.1 Traumatic spinal cord injury

**Figure 6:**
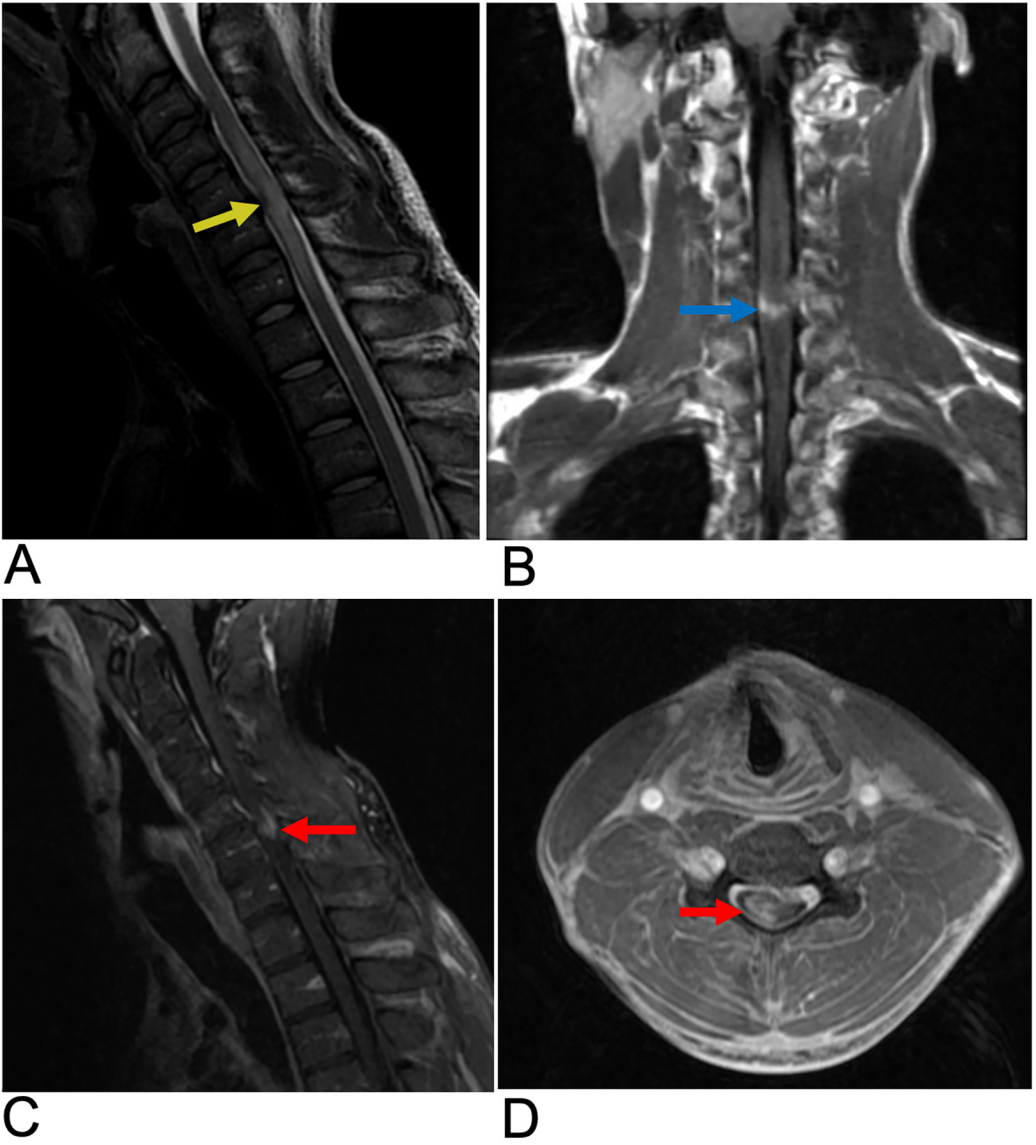
Spinal cord injury. Sagittal T2 PROP **(A)**, coronal T1 FLAIR + C **(B)**, sagittal T1 FLAIR FAT + C **(C)**, and axial 3D FSPGR Fat + C **(D)** MRI of the cervical spine showing C5-C6 cord compression (yellow arrow) resulting from posterior disc/osteophyte complex herniation. There is associated enhancement of the right hemi-cord (blue arrow) in the “pancake configuration” (red arrows) representing compressive myelopathy

Spinal cord injuries (SCI) are most often caused by blunt trauma, attributed mainly to motor vehicle accidents, followed by falls and sport injuries (40). Cervical spine injuries are the leading cause of spinal cord trauma, accounting for the majority of cases. Approximately, half of all spinal cord injuries result in significant neurological deficits (40). The economic impact of SCI is substantial with the estimated lifetime economic burden per individual with SCI ranging from $1.5 million to $3.0 million, reflecting the significant burden these injuries place on healthcare systems (41). Clinically, the presence of edema without hemorrhage in the spinal cord is generally associated with a good prognosis for recovery, as the neural tissue remains largely intact. In contrast, the development of a hematoma within the spinal cord indicates a poor prognosis, often leading to irreversible damage with minimal to no recovery. Key predictors of neurologic recovery after traumatic cervical cord injury include the extent of intramedullary hemorrhage and the degree of cord swelling, both of which directly correlate with the severity of the neurological deficit (42). MRI is the gold standard in characterizing SCI lesions. A common finding is a swollen spinal cord with a T2 hyperintense signal, reflecting cord edema caused by increased water content within the damaged tissue. This hyperintensity often extends over multiple segments, and its extent is closely associated with the severity of neurological impairment and the potential for recovery. In contrast, cord hemorrhage appears as low-intensity foci within the edematous region on T2-weighted MRI, caused by hemosiderin deposition, which creates signal voids (Figure 6) (43).

#### 2.2.2 Syringomyelia

**Figure 7:**
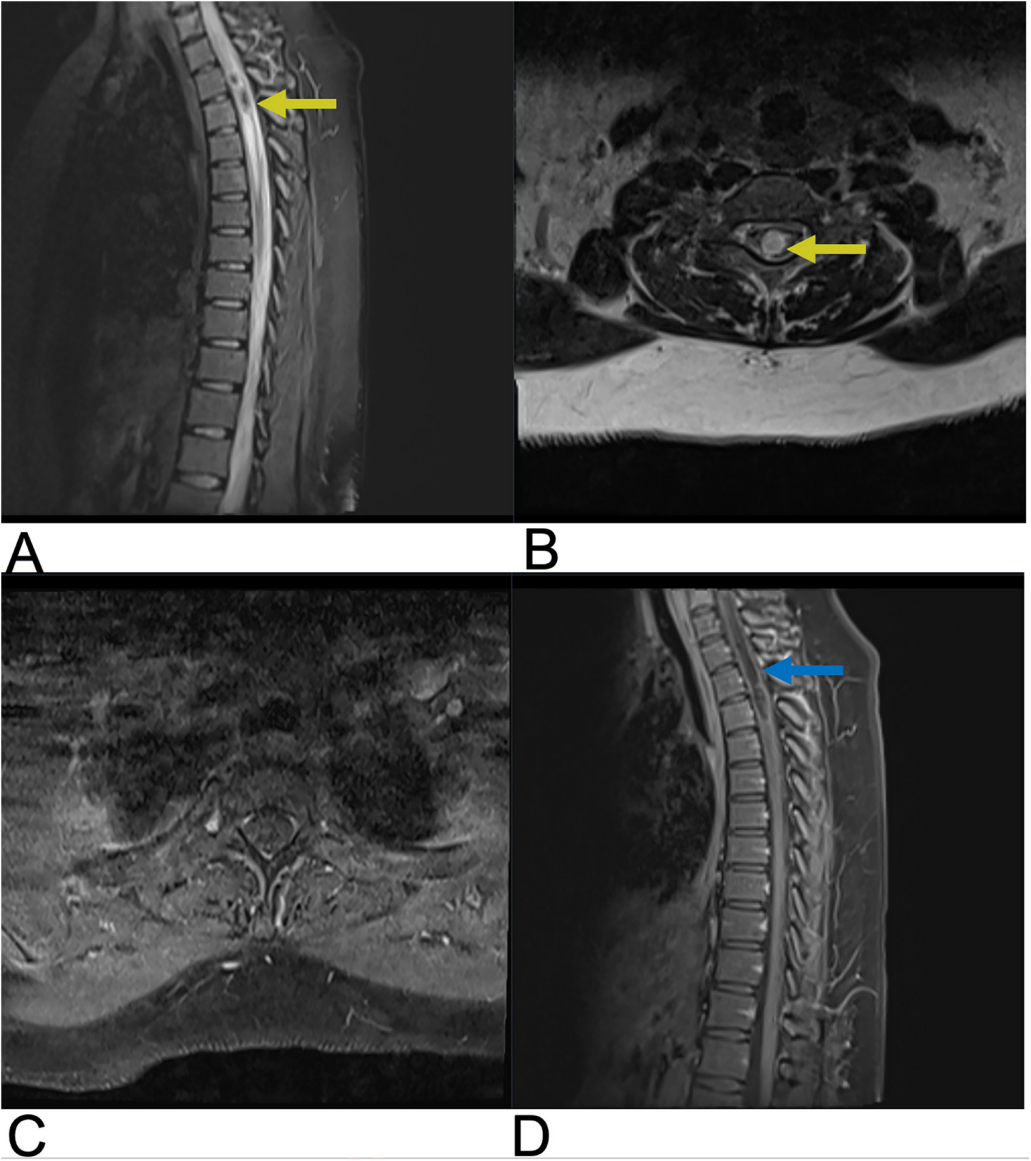
Syringomyelia. Sagittal T2 **(A)**, axial T2 **(B)**, axial **(C)**, sagittal T1 + C **(D)** MRI of the cervical and thoracic spine demonstrating a syrinx within the cervical spinal cord presenting as a hyperintense cystic cavity extending through multiple spinal segments (yellow arrows). There is edema surrounding the dilated syrinx reflecting altered CSF dynamics and cord expansion. Axial MRI illustrates a rounded, cystic cavity in the spinal cord, appearing hyperintense. The syrinx does not significantly enhance on post-contrast T1WI (blue arrow).

Syringomyelia is a disorder characterized by defective cerebrospinal fluid (CSF) circulation, culminating in the formation of fluid-filled cavities (syrinx). A syrinx is the presence of a fluid-filled cavity within the spinal cord parenchyma or central canal. The spinal cord appears enlarged due to the dilated, beaded, or sacculated cystic cavity which disrupts the normal spinal cord structure (44). Syringomyelia is usually associated with Chiari 1 or 2 malformation which obstructs CSF flow at the foramen magnum, but may also be associated with spinal cord tumors, trauma, or infectious adhesive arachnoiditis (45). While syringomyelia clinically presents with chronic pain and temperature insensitivity, the diagnosis is often made incidentally (46). Although often associated with a fully developed syrinx, this lesion can also present in a “presyrinx” state. This is a reversible condition characterized by spinal cord edema without the formation of a true syrinx and does not yet involve frank cavitation. Symptoms can potentially be reversed if the underlying cause of CSF flow disturbance is addressed promptly (47). In terms of diagnosis, MRI is the preferred imaging modality as it clearly reveals the location, size, and extent of the syrinx cavity within the spinal cord. MRI can also assess associated anatomical changes, such as the degree of cerebellar tonsillar ectopia commonly seen in Chiari malformation type I (CM1), in which the cerebellar tonsils descend below the foramen magnum. Additionally, MRI can detect the compression of retro-cerebellar CSF spaces, which contributes to syrinx formation (Figure 7) (48).

#### 2.2.3 Spinal cord infarction

**Figure 8:**
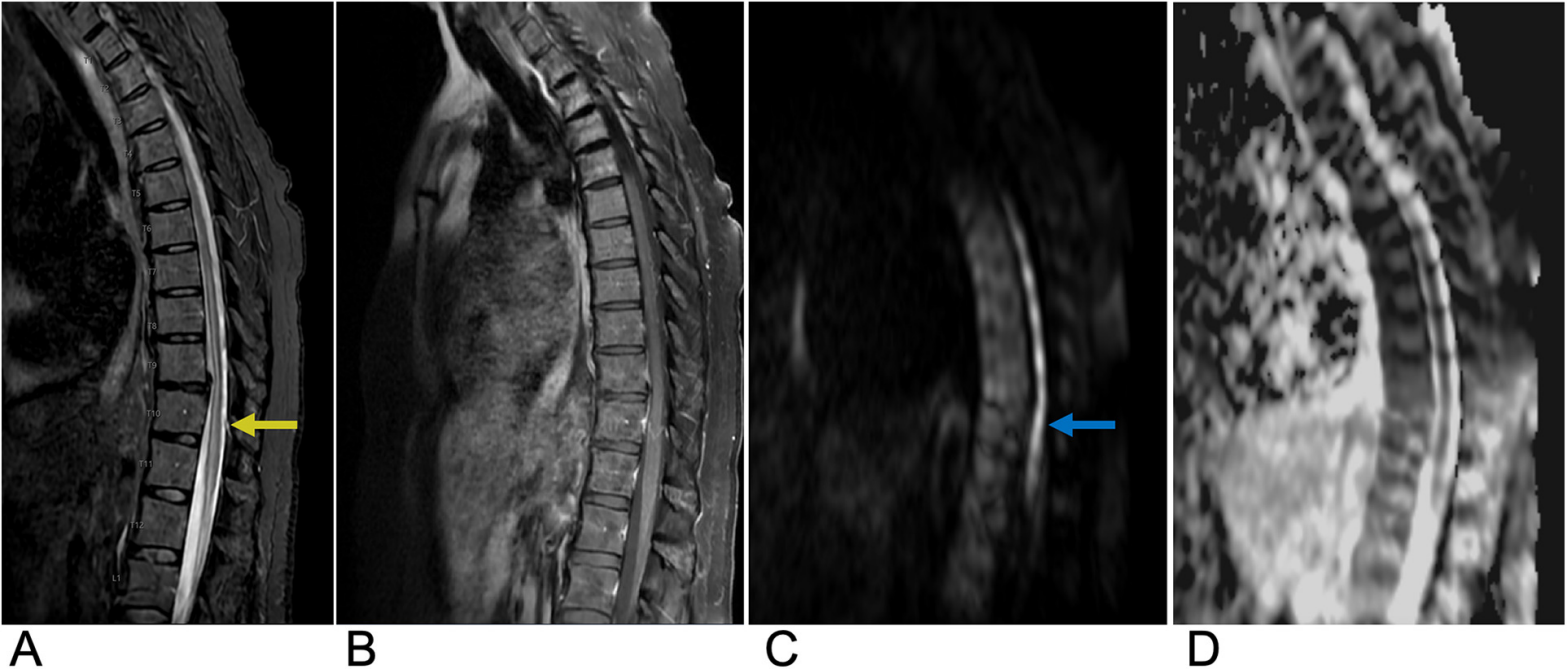
Spinal cord infarction. Sagittal T2 **(A)**, sagittal T1 FAT + C **(B)**, sagittal DWI **(C)**, and sagittal ADC **(D)** MRI of the thoracic spine demonstrating a long segment of abnormal restricted diffusion (blue arrow) from approximately T5 to the conus medullaris with associated central cord T2 signal abnormality (yellow arrow) extending from T5 through the conus medullaris concerning for spinal cord infarct.

A spinal cord infarct is a rare vascular lesion caused by a disruption of blood flow, leading to tissue death or necrosis. This condition is analogous to a stroke that occurs in the spinal cord. Its incidence rate is 3.1 per 100,000 individuals in the United States and accounts for approximately 1.2% of all strokes, primarily affecting people aged 50 to 70 years (49, 50). Anterior spinal artery syndrome (ASAS) is the most common cause, accounting for up to 87.2% of these infarcts. It results from ischemia of the anterior spinal artery and leads to damage to the anterior two-thirds of the spinal cord, affecting areas such as the spinothalamic and corticospinal tracts (51, 52). Clinically, patients present with paraparesis or quadriparesis, deficits in pain and temperature sensation, and autonomic dysfunction. In children, it is often associated with trauma or cardiac malformations, while in adults, it is associated with atherosclerosis-related conditions such as thoracoabdominal aortic aneurysms or thromboembolism (53). Diagnosis is based on clinical presentation and confirmed by MRI. Core sequences include sagittal and axial T2-weighted images to detect cord hyperintensities, often with an “owl eye” sign indicating extensive involvement (Figure 8). DWI is crucial for detecting early ischemia, revealing restricted diffusion even when T2 images are normal. Post-contrast T1-weighted imaging may show subacute enhancement. STIR sequences help identify associated bone infarcts. When MRI is contraindicated, CT myelography or CT angiography can assess vascular malformations, while Digital Subtraction Angiography (DSA) provides detailed vascular evaluation for complex anomalies. Emerging techniques like perfusion MRI, diffusion tensor imaging (DTI), and magnetization transfer imaging show potential for assessing cord microstructure and perfusion but are not yet routine (54). Treatment mainly focuses on addressing the underlying cause. While acute thrombolysis could be considered, there is insufficient evidence to support its routine use. The prognosis is poor, especially if extensive regions are involved, as patients generally exhibit long-term sequelae such as urinary catheterization and mobility difficulties. However, patients with smaller infarcts and intact proprioceptive sensation generally tend to have better outcomes. Several studies also suggest that around 50% of survivors may regain the ability to walk again, and about 50% may achieve normal urinary function (55).

#### 2.2.4 Transverse myelitis

**Figure 9:**
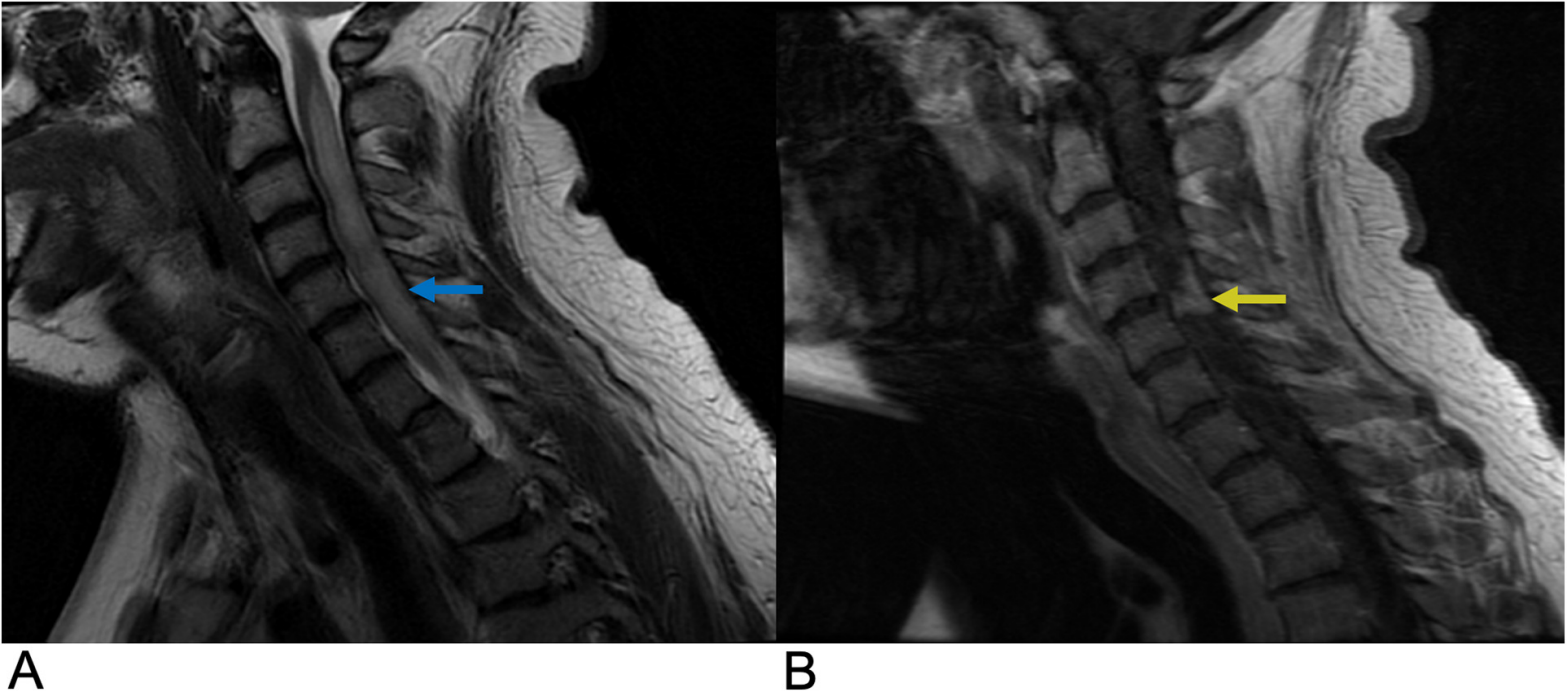
Transverse myelitis. Sagittal T2 **(A)** and sagittal T1 + C **(B)** MRI demonstrating a long segment of T2 (blue arrow) and T1C hyperintense signals (yellow arrow) occupying greater than two-thirds of the cross-sectional area of the cord representing transverse myelitis.

Transverse myelitis (TM) is an acute inflammatory disease of the spinal cord affecting both the ventral and dorsal regions. It is characterized by rapidly progressive bilateral sensory, motor, or autonomic dysfunction (56). TM can occur independently or as part of other conditions, such as neuromyelitis optica spectrum disorder (NMOSD), acute flaccid myelitis, multiple sclerosis (MS), or systemic autoimmune diseases like Sjögren's syndrome. While it can occur in any region, the thoracic spinal cord is most affected. The incidence is approximately 1–4 cases per million people annually, with peaks in individuals aged 10–19 and 30–39 (56). Symptoms may develop within hours to days and usually present with para- or tetra-paresis, sensory impairment, and sphincter dysfunction. Diagnosis is based on a combination of clinical evaluation, MRI, cerebrospinal fluid (CSF) analysis, and blood tests. MRI is the preferred imaging modality, typically revealing a long segment (>3 vertebral segments) of T2 hyperintensity that occupies more than two-thirds of the spinal cord's cross-sectional area, often with variable enhancement and no diffusion restriction (Figure 9) (55). CSF analysis commonly shows mild pleocytosis and elevated protein levels, while blood tests help rule out infections and autoimmune disorders, with Anti-NMO and Anti-MOG antibodies aiding in diagnosis (57). Electrophysiological tests, such as somatosensory evoked potentials, are used to assess spinal cord function. Emerging diagnostic trends include the use of biomarkers and advanced imaging techniques, while treatment approaches are shifting toward precision medicine, immunomodulatory therapies, and stem cell therapy (58). Although no exact cause has been identified, possible etiologies include viral infections, autoimmune conditions, and post-vaccination responses. Pathological findings typically reveal perivascular lymphocytic infiltrates, necrosis, and demyelination (59). Diagnosis and treatment vary and usually involve intravenous glucocorticoids like methylprednisolone or dexamethasone for 3 to 5 days in idiopathic cases (60). The prognosis also varies from symptoms lasting 3 to 6 months to permanent disability. Approximately one-third of patients experience minimal sequelae, another third experience moderate disability, and the remaining third suffer from severe disabilities (61).

#### 2.2.5 Demyelinating disease

Demyelinating disorders are a subtype of white matter abnormalities that involve damage to the myelinated structures of the central nervous system. These disorders can be ischemic, toxic, or inflammatory, and include conditions such as multiple sclerosis (MS), neuromyelitis optica spectrum disorder (NMOSD), progressive multifocal leukoencephalopathy (PML), and subacute sclerosing panencephalitis (SSPE). Multiple sclerosis is the most common autoimmune chronic inflammatory demyelinating disease of the central nervous system and primarily affects young adults, with peak incidence around age 35 (62). Its incidence is lower near the equator (approximately 15 per 100,000) and increases with latitude (up to 250 per 100,000), with a female predominance (approximately a 2:1 ratio). Around 12% of MS cases present with only spinal lesions (62).

Symptoms of MS can vary and may include optic neuritis, sensory disturbances, motor deficits, and cognitive decline. The condition is believed to arise from a cell-mediated autoimmune response against myelin, leading to the loss of oligodendrocytes and subsequent axonal degeneration. Demyelination typically occurs in distinct perivenular plaques that evolve through active, subacute, and chronic stages (62).

**Figure 10:**
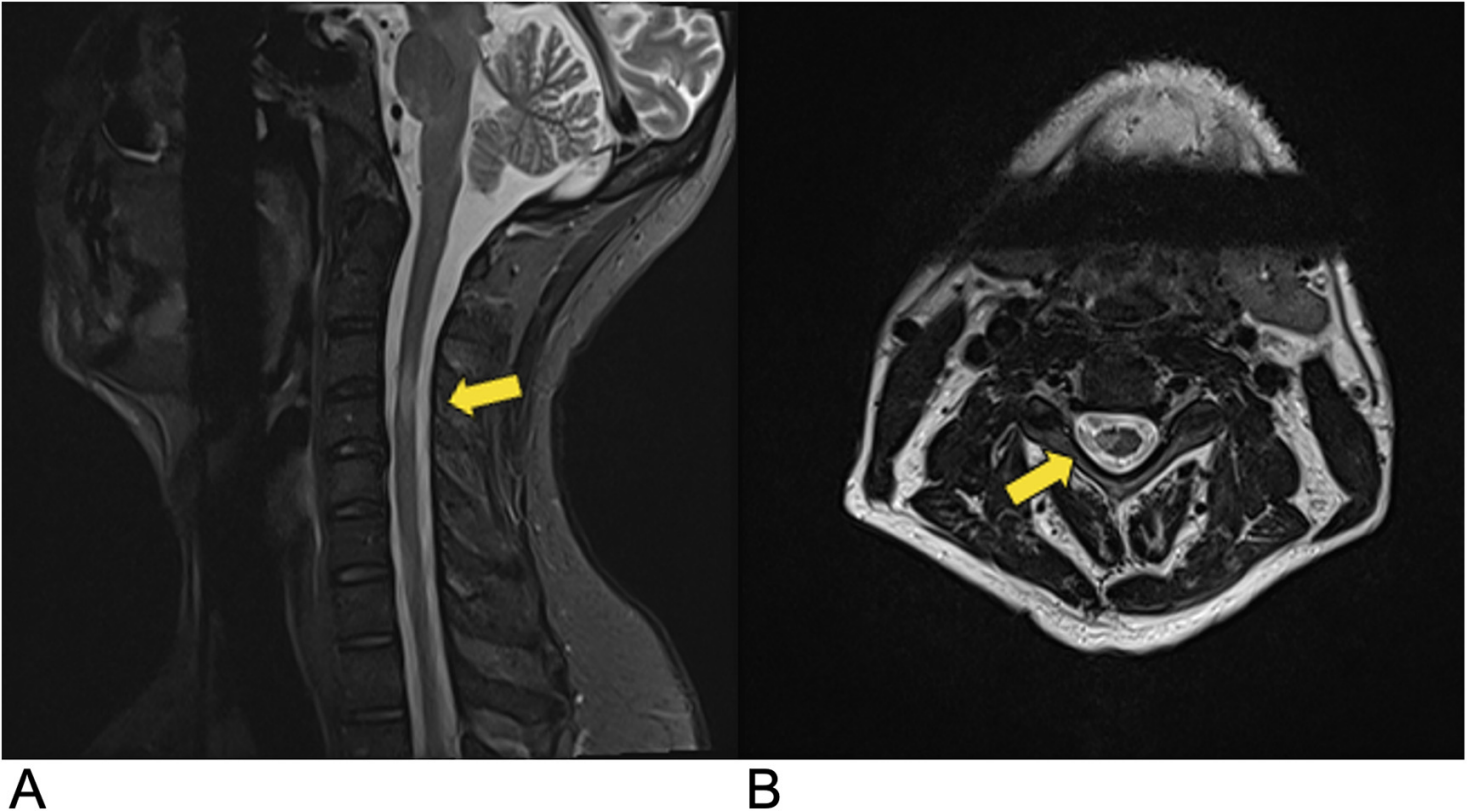
Demyelinating disease. Sagittal **(A)** and axial **(B)** T2-weighted MRI demonstrating multiple demyelinating plaques in the spinal cord (yellow arrows) consistent with multiple sclerosis.

MS presents in multiple forms, with relapsing-remitting MS (RRMS) accounting for 70% of cases and being more common in pediatric-onset MS (98%). Approximately 85% of RRMS patients develop secondary progressive MS (SPMS), while primary progressive MS (PPMS) is rare (62, 63). The diagnosis of multiple sclerosis (MS) is based on clinical presentation, MRI findings, cerebrospinal fluid (CSF) analysis, and electrophysiological tests. MRI typically reveals scattered, wedge-shaped, T2 hyperintense lesions larger than 3 mm, located peripherally and extending less than three vertebral levels. Active lesions may enhance with gadolinium contrast (Figure 10). CSF analysis often shows the presence of oligoclonal bands, and visual evoked potentials may be used to assess optic nerve involvement (64). Recent trends in MS diagnosis focus on the use of advanced MRI techniques, biomarkers for early detection, and machine learning models for automated lesion detection. Future directions involve precision medicine, including personalized treatment strategies based on genetic and immunological profiles (65) (Figure 10). Genetic and environmental factors, including a potential association with HLA-DR15 and Epstein–Barr virus (EBV), may also contribute to the disease (62). Prognosis varies depending on the disease pattern and severity, and treatment primarily focuses on managing symptoms and employing disease-modifying therapies.

### 2.3 Spinal intradural-extramedullary tumors

Intradural-extramedullary spinal metastases (IESMs) are relatively rare, accounting for ≤5% of all metastases. However, IESM incidence is on the rise due to advancements in cancer treatment, longer patient survival rates, and improvements in diagnostic imaging (66). IESMs are the second most common type of intradural-extramedullary tumors, usually arising from the leptomeninges or nerve roots, and are primarily associated with cancers of the lung, breast, and melanoma. The main intradural-extramedullary neoplasms include meningioma (20%–30%) and schwannoma (15%–50%), while neurofibroma include rarer types, such as solitary fibrous tumors/hemangiopericytoma and malignant peripheral nerve sheath tumors (67). Leptomeningeal metastases, seen in 5%–15% of solid tumors, are commonly linked to melanoma, small cell lung cancer, and breast cancer (67). Clinically, patients present with nocturnal pain, motor and sensory dysfunction, reflex abnormalities, long tract signs, and autonomic dysfunction. MRI is the preferred imaging modality, typically revealing a mass within the spinal canal that may extend into the neural foramina and paraspinal regions, often indicating a nerve sheath tumor. When imaging provides adequate contrast resolution of the cerebrospinal fluid (CSF) space, the mass is usually localized within the dura but outside the spinal cord (66, 67). Diagnosis may require histopathological assessment. Future trends may focus on the integration of AI-driven diagnostic tools for more accurate and faster detection, as well as intraoperative MRI guidance to improve surgical outcomes (68). Treatment involves surgical removal, which is often successful since these tumors are typically benign. The growing incidence of these diagnoses highlights the need for increased awareness and precise imaging to ensure optimal patient management.

#### 2.3.1 Meningioma

**Figure 11:**
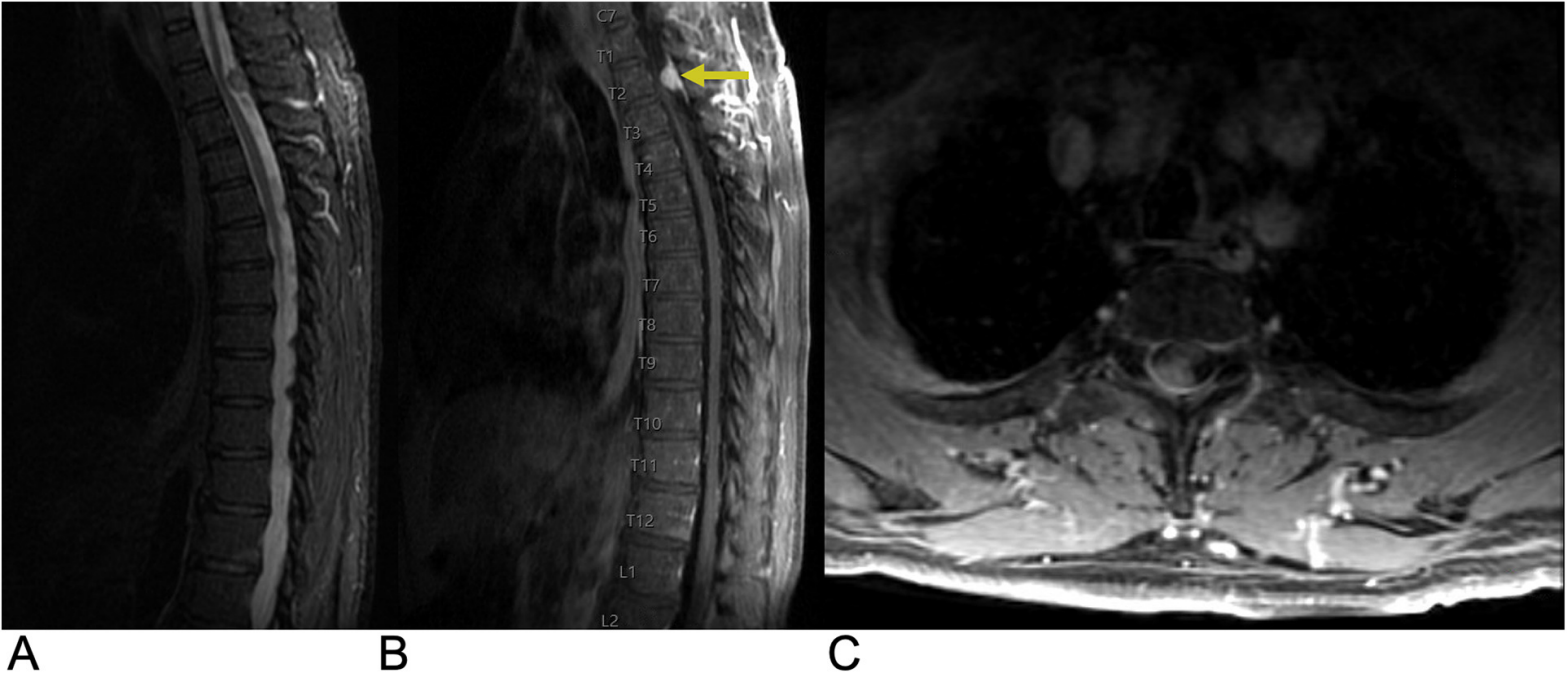
Meningioma. Sagittal T2 STIR **(A)**, sagittal T1 FLAIR FAT + C **(B)**, axial T1 FAT + C **(C)** MRI of the thoracic spine demonstrating an intradural, extramedullary oval-shaped enhancing lesion (yellow arrow) representing a spinal canal meningioma involving the right dorsolateral aspect of the thecal sac at the T2 level with moderate narrowing of the spinal canal.

Meningiomas account for around 36% of all primary central nervous system (CNS) tumors and 53% of non-malignant CNS tumors, making them the most common type of primary CNS tumor. They have an incidence rate of 7.86 cases per 100,000 people (69). Spinal meningiomas represent approximately 12% of all meningiomas, with peak incidence occurring between the sixth and eighth decades of life (70). They show a strong female predominance, with women comprising 75%–90% of adult cases (M:F ratio of 1:4), though there is no observed sex bias in children, and they are more common in adults than in children. Most spinal meningiomas (90%) are intradural-extramedullary, predominantly located laterally to the spinal cord, with the thoracic spine being the most common site (80%) (71). While the majority of spinal meningiomas are benign and classified as WHO grade I (70%–90%), less common variants may exhibit more aggressive features (WHO grade II: 5%–25%; grade III: 1%–5%) (67). MRI typically reveals hypointensity on T1-weighted images and hyperintensity on T2-weighted images, with the “ginkgo leaf sign” helping to distinguish them from schwannomas (Figure 11). Recent advances in imaging include perfusion imaging, fMRI, and dynamic contrast-enhanced MRI, which enhance tumor assessment, while AI and machine learning help improve diagnostic accuracy (72). Future trends include intraoperative MRI for real-time guidance, molecular imaging for personalized treatment, and radiomics combined with AI for better surgical outcomes and prognostication (73). Clinically, patients present with motor deficits due to spinal cord compression, as well as less common symptoms such as sensory deficits, pain, and sphincter dysfunction. Environmental risk factors include exposure to high-dose ionizing radiation and prior trauma, while genetic conditions like neurofibromatosis type 2 (NF2) significantly increase incidence, particularly in pediatric patients. Surgery is the preferred treatment, with complete removal achieved in the vast majority of cases and a recurrence rate of less than 10% (74).

#### 2.3.2 Schwannoma

**Figure 12:**
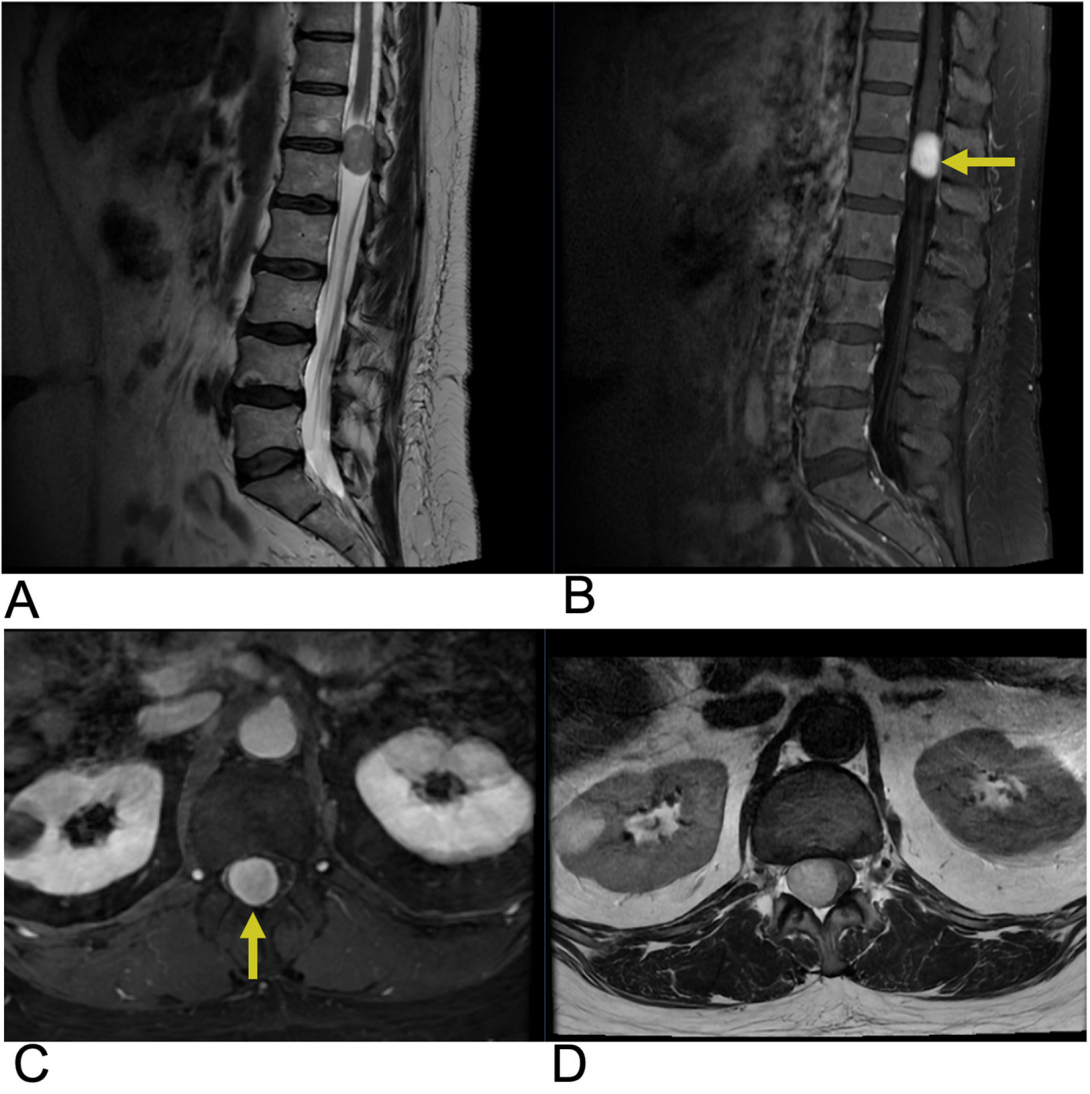
Schwannoma. Sagittal T2 **(A)**, sagittal T1 + C **(B)**, axial T1 + C, axial T2 **(D)** MRI of the lumbar spine demonstrating an intradural, extramedullary enhancing foci (yellow arrows) at the T12 level representing a biopsy-proven schwannoma causing mass effect affecting the spinal cord.

Schwannomas, also known as neurilemmomas, are benign tumors that arise from the myelin sheath of peripheral nerves. These slow-growing tumors are composed exclusively of Schwann cells, which produce the myelin that insulates nerve fibers. Schwannomas represent about 8% of all nervous system tumors and are the most common type of nerve sheath tumor, accounting for around 89% of such cases (75). They can occur sporadically or in conjunction with Neurofibromatosis type 2 (NF2) (76). While 95% of spinal schwannomas are solitary or sporadic, nearly all spinal nerve root tumors in NF2 patients are either schwannomas or mixed tumors (67). The incidence of schwannomas peaks between the fifth and seventh decades of life, and they do not show a sex preference. Clinically, patients present with symptoms of pain and radicular sensory changes, while weakness is less frequent, and large tumors can lead to myelopathy. Imaging protocols for schwannoma have evolved with advancements in MRI techniques. Imaging typically reveals an oval or round mass with isointense or hypointense signals on T1-weighted MRI and hyperintense, heterogeneous signals on T2-weighted images. These tumors often appear as well-circumscribed, dumbbell-shaped transforaminal masses, primarily affecting the cervical and lumbar regions, where they originate from dorsal sensory roots. Recent advancements in high-resolution MRI, including 3 T imaging and diffusion tensor imaging, enhance tumor characterization and aid in surgical planning. Future trends may involve incorporating functional imaging and molecular biomarkers to improve diagnosis and treatment strategies (77). Pathologically, schwannomas are well-defined, globular lesions that are often encapsulated and distinct from surrounding nerve roots (Figure 12) (75). Schwannomas grow slowly and rarely transform into malignant tumors, so surgical resection is generally the preferred treatment, with gross total resection often being curative for sporadic cases. However, patients with NF2 have a high likelihood of developing new tumors (78).

#### 2.3.3 Neurofibroma

Neurofibromas are benign peripheral nerve sheath tumors that are primarily composed of Schwann cells, fibroblasts, myxoid material, and peripheral nerve fibers. They can present in different forms, such as localized, diffuse, and plexiform types, with plexiform neurofibromas (PNF) notably linked to Neurofibromatosis type 1 (NF1). These tumors can develop in the intradural, extramedullary, or extradural/paraspinal compartments. Malignant transformation to malignant peripheral nerve sheath tumors (MPNSTs) occurs in approximately 10% of NF1 patients with plexiform neurofibromas (79).

**Figure 13:**
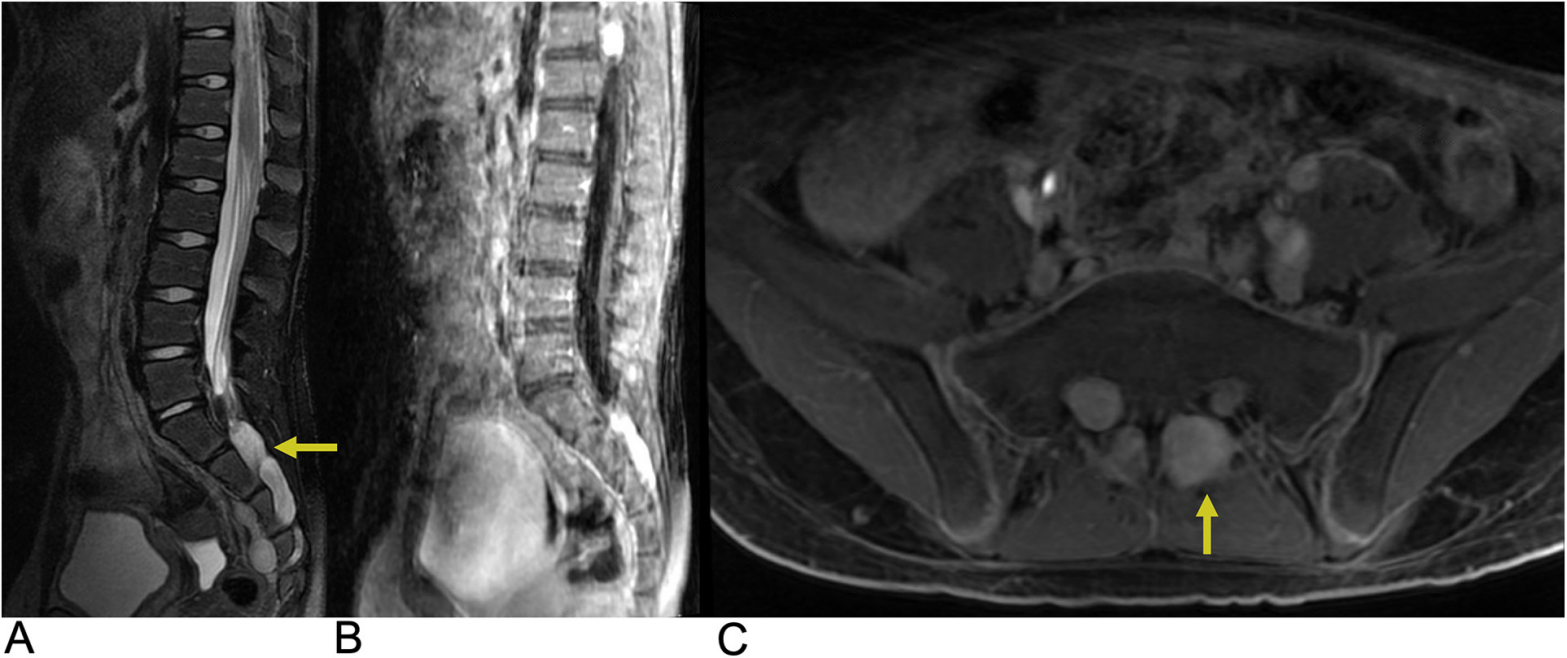
Neurofibroma. Sagittal T2 FLAIR **(A)**, sagittal T1 FLAIR + C **(B)**, and axial T1 FAT + C **(C)** MRI demonstrating numerous neurofibromas (yellow arrows) throughout the lumbosacral spine with involvement of the L3–L4 and S1–S4 levels along the greater sciatic notch with associated remodeling of the neural foramina.

Spinal neurofibromas often occur along the dorsal sensory roots and are typically asymptomatic. Clinically, patients may present with pain, radicular sensory changes, or weakness (80). Multiple lesions can lead to radiculopathies, and larger tumors may cause myelopathy (80). MRI studies generally reveal spinal neurofibromas with similar intensity to the spinal cord and nerve roots on T1-weighted images and iso- or hyperintensity on T2-weighted images, often displaying a characteristic “target sign”. These tumors can also cause remodeling of adjacent bones, leading to widening of the neural exit foramen and scalloping of the vertebrae (Figure 13). Future trends may incorporate the use of molecular imaging and artificial intelligence (AI) to further improve diagnostic accuracy and assist in treatment planning (81). While surgery is the preferred treatment for symptomatic lesions, complete dissection from nerve roots can be difficult. Approximately 5%–10% of neurofibromas may undergo malignant transformation, which is typically signaled by rapid growth (80).

## 3 Lesions involving the epidural space

### 3.1 Epidural abscess

**Figure 14:**
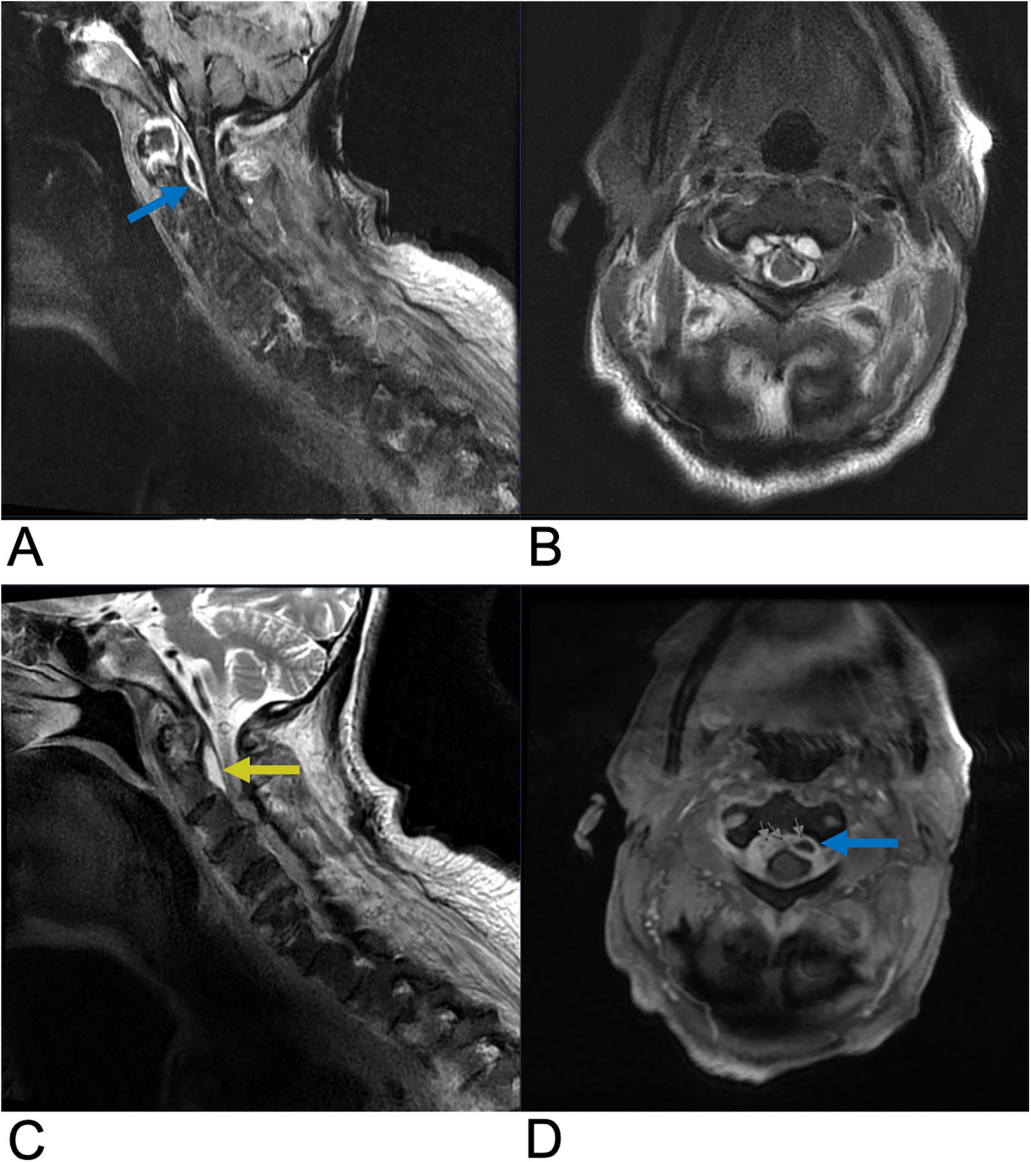
Epidural abscess. Sagittal T1 + C **(A)**, axial T2 **(B)**, sagittal T2 **(C)**, and axial T1 + C **(D)** MRI demonstrating a T2 hyperintense lesion (yellow arrow) at the epidural space encroaching on the spinal canal and causing spinal cord compression. The abscess is peripherally enhancing on post-contrast T1 weighted MRI (blue arrows) consistent with surrounding inflammatory tissue.

The spinal epidural space is located between the dura mater and vertebral bone, containing an intricate venous network (82). This venous network, called the Batson Plexus, facilitates the venous drainage of the spinal cord and epidural space, often communicating with systemic circulation. The plexus contains no valves and moves with a bidirectional flow (82, 83). Thus, there is high potential for the epidural space to become infected via hematogenous or contiguous spread of infections such as discitis, osteomyelitis, or paraspinal abscess (82, 83). Bacterial infections within the epidural space are most common (84, 85). Spinal epidural abscess is a rare etiology with debilitating consequences, such as permanent neurological decline, paralysis, and even death if left untreated (82). Incidence of spinal epidural abscess has been historically low, but has been increasing in recent decades, and this rise can be attributed to multiple factors such as an aging population, growing prevalence of immunosuppressive comorbidities like diabetes mellitus, and more frequent administration of spinal procedures (82, 86, 87). These are all common risk factors that contribute to the emergence of epidural abscesses (82, 88, 89). Moreover, presentation and progression are quite variable, often making diagnosis challenging. Relying on clinical findings alone can often lead to misdiagnosis and treatment delays, and MRI plays a crucial role in reducing these delays (82, 86, 90). MRI findings of a spinal epidural abscess typically show a localized focus of high signal intensity on T2-weighted images, reflecting inflammation, edema, or fluid accumulation within the epidural space. On T1-weighted images, the abscess appears as a hypo-intense area due to the low protein content of the pus within the abscess cavity (Figure 14). After gadolinium contrast administration, there is usually a rim of peripheral enhancement, which represents the vascularized inflammatory tissue surrounding the abscess. The central portion of the abscess, filled with pus, may not enhance (89). The abscess may cause compression of adjacent structures, such as the spinal cord or nerve roots, depending on its size and location, leading to neurological symptoms like weakness or sensory deficits. Additionally, there may be signs of surrounding soft tissue inflammation or involvement of adjacent structures, such as vertebral osteomyelitis or discitis, which can be indicated by abnormal signal intensity in the vertebral bodies or intervertebral discs (89).

### 3.2 Epidural hematoma

Spinal hematoma is a rare neurological disorder that may lead to death or severe neurologic deficit without treatment. In a review of 613 spinal hematoma cases, 29.7% of patients presented without identifiable etiologic factors (91). Known etiologic factors for spinal hematoma include anticoagulant therapy, vascular malformations, trauma, and spinal anesthetic procedures. Spinal hematomas are most common in patients aged 55–70 and men comprise 63.9% of evaluated patients (91). In 2.1% of cases, spinal hematoma may involve multiple meningeal compartments. Epidural hematoma, which is the most common form of spinal hematoma, involves the space between the dura mater and vertebral bodies. Epidural and subdural hematoma may characteristically present with “coup de poignard”, which is an intense pain localized to the region of hemorrhage, followed by a pain-free interval lasting minutes to days and subsequent paralysis below the affected spinal level (91).

Epidural hematoma may be more common than subdural hematoma in the spine due to the lack of bridging veins in the spinal subdural potential space (92). Management of epidural hematoma depends on etiology, presence of comorbidities such as coagulopathy, and level of neurological impairment due to spinal cord impingement. Patients presenting with very mild neurologic deficits or those who exhibit early spontaneous improvement may be managed conservatively, while those presenting with more severe neurologic impairment due to cord compression may require surgical decompression (92). The highest recovery rates are seen in patients who receive surgery within 12 h of symptom onset with 39.6% of evaluated spinal hemorrhage patients making a full recovery (91).

**Figure 15:**
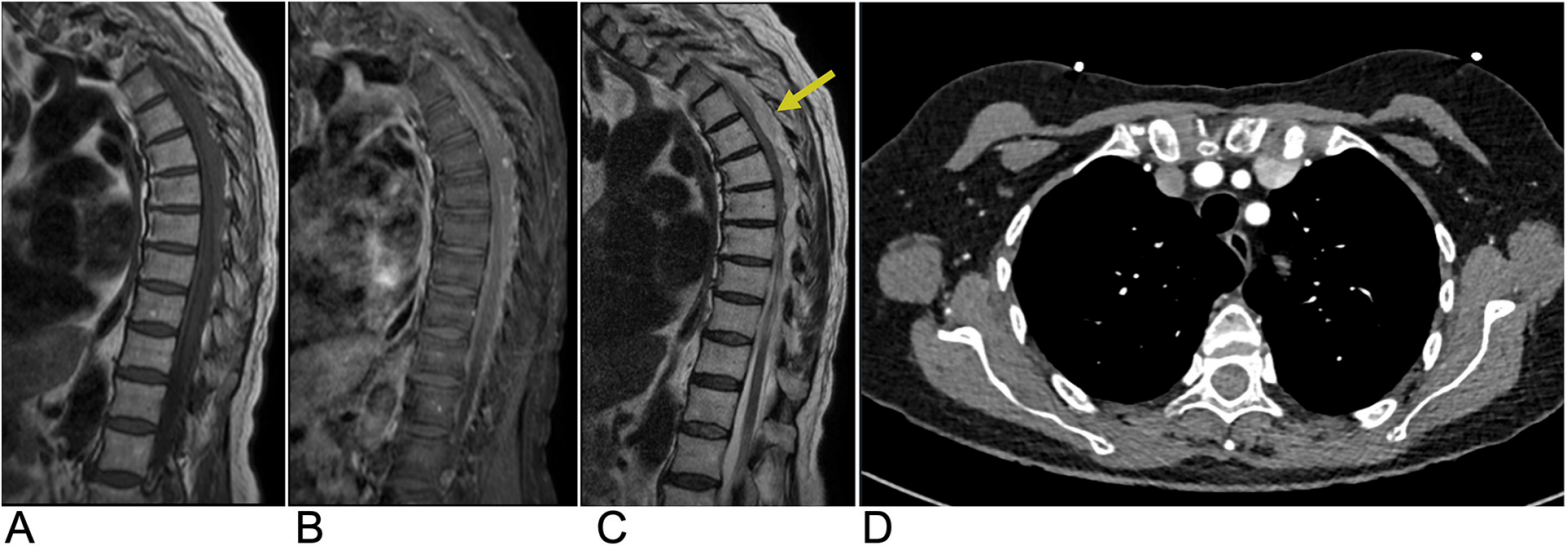
Epidural hematoma. Sagittal T1 **(A)**, sagittal T1 + C **(B)**, and sagittal T2 **(C)** MRI as well as an axial CT **(D)** of the thoracic spine demonstrating a T1 isointense, T2 hyperintense (yellow arrow), and CT isodense collection in the dorsal epidural space of the midthoracic spine representing epidural hematoma with associated mass effect on the spinal cord.

When spinal hematoma is suspected, early MRI in multiple planes is crucial to determine the location and extent of the hemorrhage as well as concurrent spinal injuries. Spinal hematoma has fluctuating characteristics on MRI from hyperacute to acute and early-late subacute phases (92). In the hyperacute phase, spinal hematomas are isointense on T1-WI and hyperintense on T2-WI with a rim of hypointensity being possible. In the acute phase, these lesions appear hypo-isointense on T1-WI and hypointense on T2-WI due to intracellular deoxyhemoglobin. In the early subacute phase, hyperintensity on T1-WI and hypointensity on T2-WI can be seen, while the late subacute phase shows hyperintensity on both T1-WI and T2-WI due to extracellular methemoglobin. Chronic stage hematoma appears hypointense on both T1-WI and T2-WI due to hemosiderin and ferritin accumulation (92). Epidural hematoma may be localized by the loss of normal epidural fat signal on MRI with a smooth contour adjacent to the spinal cord due to the containment of the hematoma by the dura mater (Figure 15). On T2-WI, a hypointense line surrounding the spinal canal may be seen which indicates bulging of the dura into the spinal canal due to displacement by the epidural hematoma. For conservatively managed patients, follow-up MRI is important to determine whether the lesion has resolved or requires further management (92).

### 3.3 Epidural tumor

**Figure 16:**
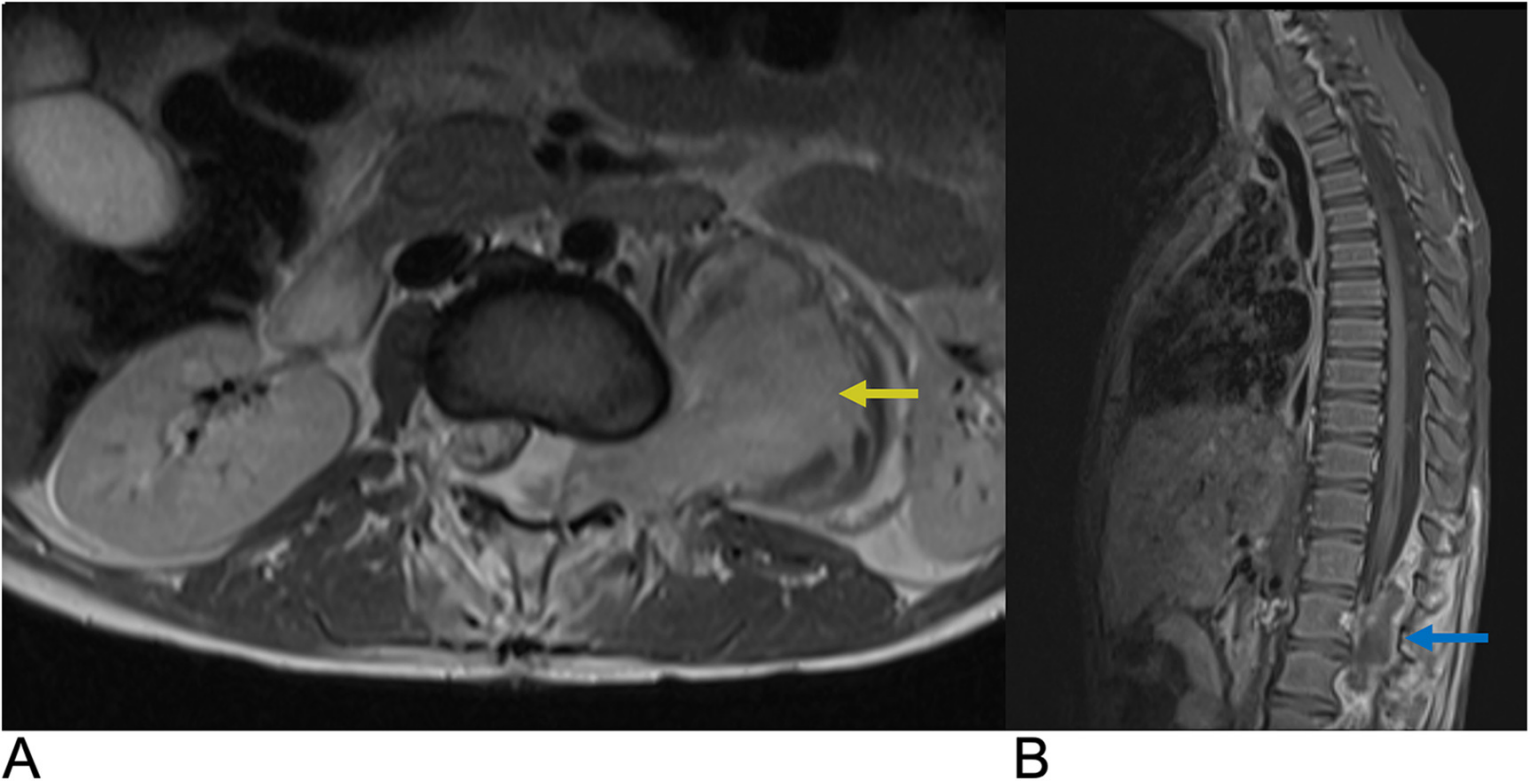
Epidural tumor. Axial T1 + C **(A)** and Sagittal T1 + C **(B)** MRI demonstrating an enhancing epidural malignant lesion (blue arrow) extending laterally through the neural foramen with infiltration of the left psoas muscle (yellow arrow) and associated spinal cord compression.

Spinal tumors presenting in the extradural space include chordoma, Ewing sarcoma, osteosarcoma, and lymphoma, but metastases from distant sites account for the majority of epidural tumors (1, 93). Many cases of epidural metastases remain asymptomatic and are diagnosed incidentally on imaging (93). Symptomatic epidural metastases to the spine are found in 5%–10% of cancer patients and often occur via hematogenous spread from the primary tumor site (1, 93). In 5%–10% of cancer patients, epidural metastases may lead to metastatic epidural spinal cord compression (MESCC) causing progressive pain, upper motor neuron signs, and possibly irreversible neurologic deficits. Epidural metastases in the lumbosacral area can potentially lead to cauda equina compression causing bladder/bowel dysfunction, saddle anesthesia, and motor/sensory deficits of the lower extremities (93). 85% of epidural metastases originate from the vertebral column due to the presence of highly vascular bone marrow allowing for hematogenous tumor spread to the vertebral bodies and to a lesser extent, the vertebral arches. These bony metastases can grow into the epidural space overlying the dura mater leading to cord compression, vasogenic edema due to the occlusion of venous drainage, and spinal cord infarction (93). In addition, 10%–15% of epidural metastases originate from paraspinal soft tissue and may spread to the epidural space through the vertebral foramina. The most common regions for epidural metastases are in the thoracic spine (60%) as well as lumbosacral and cervical spine (10%), while multi-level spinal involvement is seen in 20%–35% of cases (93). Management of epidural metastases is time-sensitive and depends on early recognition of MESCC to limit the extent of irreversible spinal cord damage (94). T1 and T2 weighted MRI of the spine with contrast is indicated when MESCC is suspected due to the potential for multi-spinal level metastatic involvement in 20%–35% of patients (93). MRI allows for the evaluation of the bone involvement and extent of epidural spread as well as identification of regions of MESCC, and post-contrast MRI allows for the identification of the metastatic border (Figure 16). CT may be useful for further characterizing the extent of bony involvement and bone integrity prior to surgical planning (93).

## 4 Intervertebral disc lesions

### 4.1 Intervertebral disc degeneration

**Figure 17:**
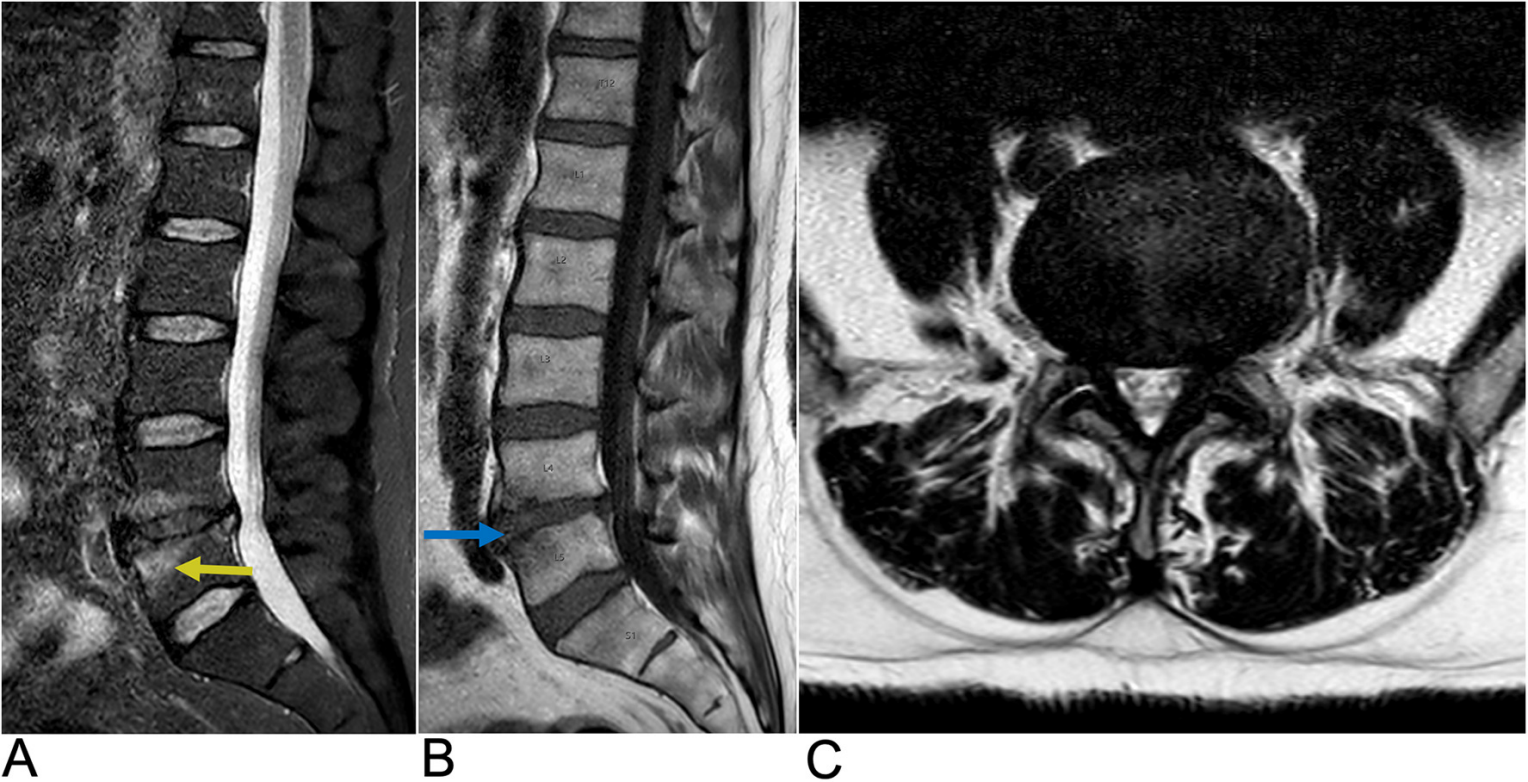
Disc degeneration. Sagittal T2 **(A)**, sagittal T1 **(B)**, and axial T2 **(C)** MRI demonstrating chronic spondylitic degenerative changes at L4/5 including diffuse disc bulge (blue arrow), loss of intervertebral disc height, and Modic Type 1 endplate degenerative changes (yellow arrow).

Intervertebral disc degeneration (IDD) is a progressive condition characterized by the degradation of intervertebral discs, which are vital for cushioning and providing structural support between vertebrae (95). It is primarily caused by advancing age, mechanical overload, and genetic factors. IDD leads to a reduction in disc height, formation of fissures in the annulus fibrosus, and a weakening of the overall structural integrity of the discs. Epidemiologically, IDD affects approximately 5% of the population in developed countries each year, with its prevalence increasing with age (96). The Wakayama Spine Study, a population-based cohort, found that the prevalence of IDD was 71% in men and 77% in women under 50, rising to over 90% in both genders over age 50. The most commonly affected intervertebral spaces were C5/6, T6/7, and L4/5 (Figure 17), with age and obesity being significant risk factors. Low back pain is frequently linked to lumbar region degeneration (96). Clinically, patients present with symptoms like lower back pain and nerve compression, potentially leading to disc herniation.

Imaging features are crucial for diagnosis, with the Pfirrmann classification system widely used on T2-weighted MRI to evaluate the degree of degeneration. This system classifies disc degeneration into five grades, with grades 4 and 5 indicating severe degeneration (97). Genetic factors also play a significant role in the progression of IDD. For example, variations in the vitamin D receptor (VDR) have been associated with differing IDD risks across ethnic groups, while the THBS2 gene has been linked to lumbar disc degeneration (98, 99). Genetic studies, including twin research, have shown that heredity accounts for up to 74% of variance in disc degeneration, with specific gene polymorphisms playing a role (100).

Histologically, degenerated discs exhibit reduced proteoglycan content in the nucleus pulposus, inflammation, and fissures in the annulus fibrosus. Diagnosis relies on clinical evaluation and imaging, with MRI being the gold standard. Treatment typically starts with conservative measures like physical therapy, NSAIDs, and lifestyle modifications. Pharmacological options, including analgesics, muscle relaxants, and corticosteroids, are often used to manage symptoms. In severe cases, surgical interventions like spinal fusion or discectomy may be necessary. Emerging therapies, such as cell-based treatments, growth factor injections, and gene therapy, are being explored to regenerate disc tissue and restore function (101, 102).

### 4.2 Disc herniation

Disc herniation occurs when the nucleus pulposus, a jelly-like substance at the core of the intervertebral disc, is displaced from its normal intervertebral space through the annulus fibrosus, compressing adjacent neural structures. This condition is particularly prevalent in individuals aged 30 to 50, with estimates suggesting that 1%–3% of the population experiences symptomatic herniation annually. Additionally, studies indicate that around 80% of people will experience lower back pain at some point in their lives, with disc herniation being a leading cause (103, 104). Clinically, patients present with symptoms such as radicular pain, neurogenic claudication, or, in severe cases, cauda equina syndrome (103, 104). Genetic factors contributing to disc herniation include variants in collagen genes (COL1A1, COL9A3, COL2A1), notably the COL9A3 rs6122316-C variant. The aggrecan gene (ACAN) variant rs3817428 is linked to severity, while the 8q24.21 variant (rs6651255) is associated with sciatica in younger patients. Additionally, polymorphisms in the ADAMTS6, ADAMTS17, and BDNF/BDNFOS genes highlight the multifactorial nature of lumbar disc herniation susceptibility (105–109).

Imaging features of herniated discs are crucial for understanding their complications and include four types: protrusion, extrusion, migration, and sequestration. Protrusions remain at the level of the disc with intact outer annular fibers. Extrusions involve a complete tear of the annulus fibrosus, allowing nuclear material to extend beyond the disc and potentially beyond the adjacent vertebrae. Migration refers to the displacement of disc material away from the site of extrusion into the epidural space without complete sequestration. Sequestration involves the separation of a herniated disc fragment from the parent disc, allowing it to float freely within the epidural space. The histopathology of disc herniation features granulation tissue, macrophage infiltration, and elevated inflammatory mediators like MMP-3 and TNF-alpha. It also includes nerve fibers associated with pain, fragments of the cartilaginous endplate, and advanced degeneration of the annulus, all contributing to clinical symptoms and disease progression (110, 111).

**Figure 18:**
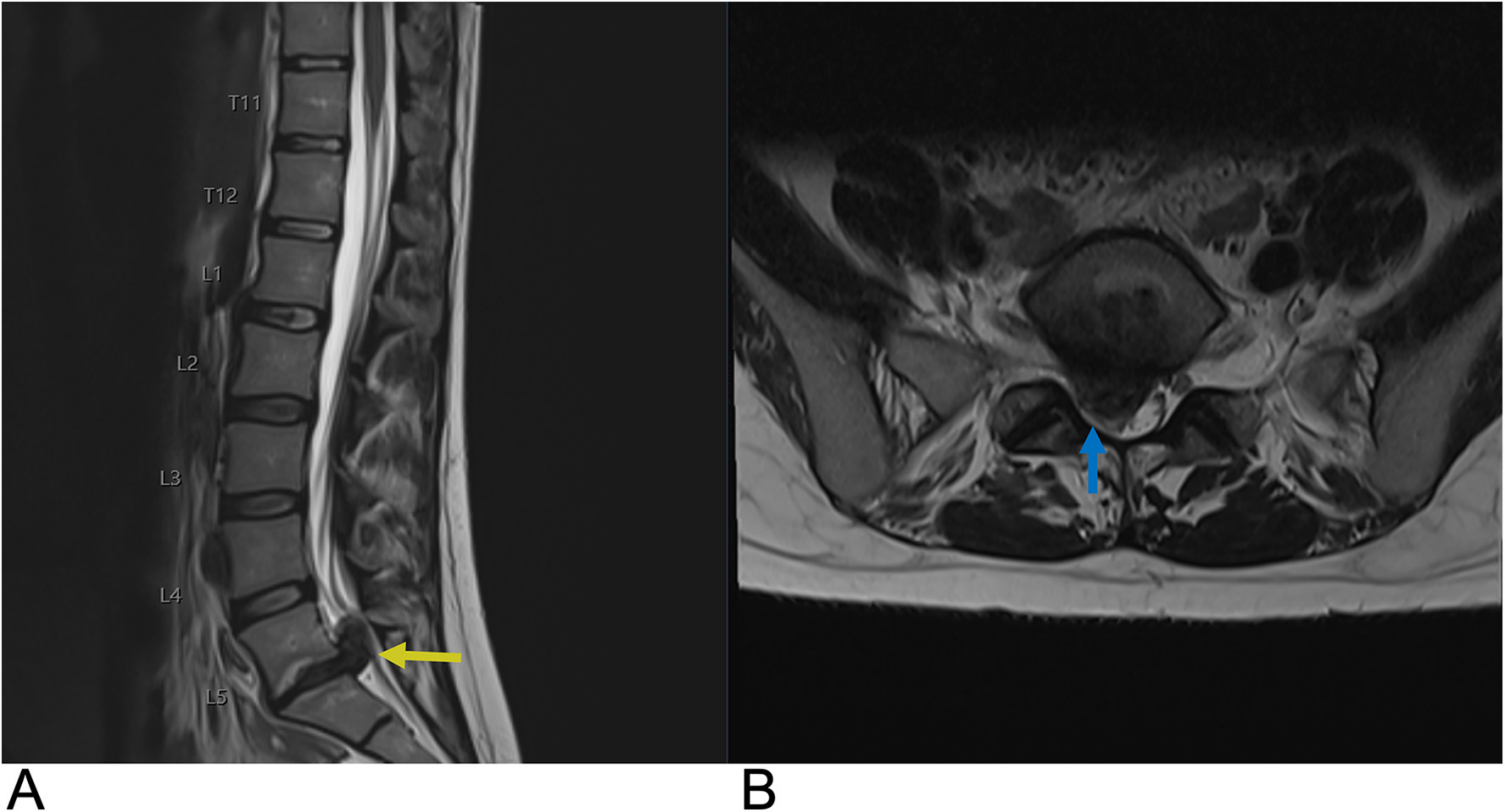
Disc herniation. Sagittal T2 **(A)** and axial T2 **(B)** MRI of the lumbar spine demonstrating a large right paracentral broad-based disc herniation at the L5-S1 level (yellow arrow) with the herniated disc pushing into the spinal canal. On axial MRI, the herniated disc can be seen causing effacement of the right subarticular zone with compression of the right S1 nerve root (blue arrow).

MRI remains the gold standard, providing detailed visualization of disc morphology and surrounding structures. It can differentiate between protrusions and extrusions based on their characteristics. For example, MRI reveals that protrusions display an intact annulus with localized disc material, while extrusions show annular tears with nuclear material extending past the endplates (Figure 18). Treatment is primarily conservative, focusing on symptom management through physical therapy and possibly epidural steroid injections. Surgical interventions, such as microdiscectomy or laminectomy, are reserved for severe cases with persistent symptoms or neurologic deficits. Overall, understanding disc herniation includes its classification, imaging characteristics, and management strategies aimed at optimizing patient outcomes (112, 113).

### 4.3 Discitis-Osteomyelitis

Discitis is an inflammation of the intervertebral disc space, often caused by infection, although non-infectious causes have been reported as well. It most commonly occurs in the lumbar region but can affect any part of the spine (114). Discitis is frequently linked to bacterial infections, with Staphylococcus aureus being the most prevalent pathogen (115). The condition can develop in individuals of any age, though it is more commonly seen in children than older adults (116). Risk factors include immunosuppression, recent spine surgery, diabetes, and intravenous drug use (117). If untreated, discitis can lead to serious complications, such as vertebral osteomyelitis and spinal abscesses, potentially resulting in neurological deficits.

The clinical presentation of discitis varies, with symptoms often including severe back pain that worsens with movement, fever, and, in some cases, neurological symptoms such as weakness or numbness in the limbs. In younger children, symptoms can be more nonspecific, such as irritability, refusal to walk, and abdominal pain. Due to its variable presentation, discitis can be challenging to diagnose, especially in the early stages (115–117).

**Figure 19:**
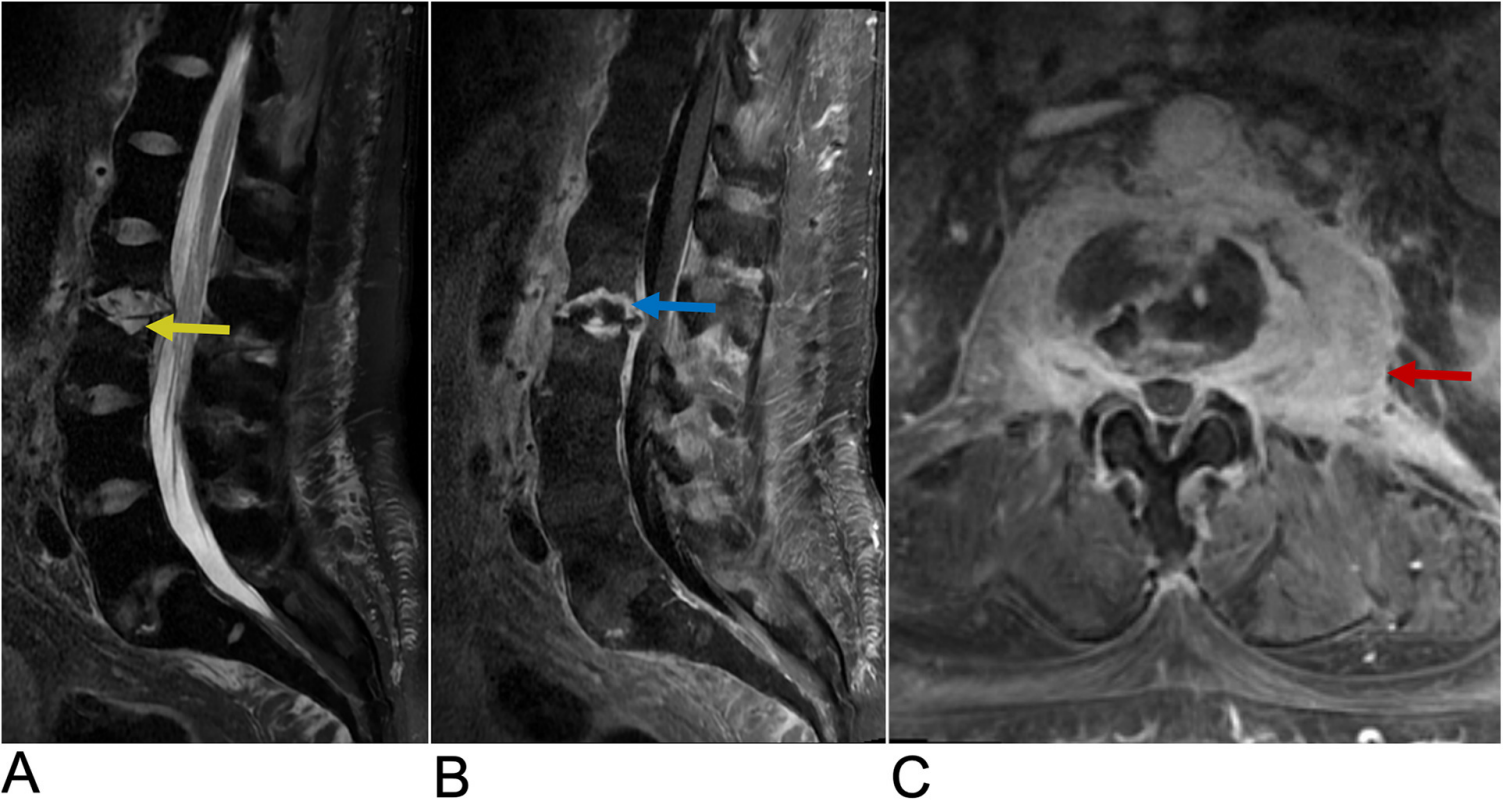
Discitis-Osteomyelitis. Sagittal T2 **(A)**, Sagittal T1 + C **(B)**, and axial T1 + C **(C)** MRI of the lumbar spine demonstrating acute discitis osteomyelitis involving the L2–L3 level with endplate erosive changes (yellow arrow), post-contrast enhancement (blue arrow), and trace fluid in the intervertebral disc space. There is posterior ventral epidural extension of the phlegmonous changes and bilateral paravertebral extension of the enhancement involving the psoas muscles bilaterally (red arrow).

MRI is considered the gold standard imaging modality for diagnosing discitis, as it can identify inflammation in the disc space, bone marrow edema, and possible abscesses. Positive imaging often reveals narrowing of the intervertebral disc space and destruction of adjacent vertebral endplates, which are indicative of infectious or inflammatory involvement (118). A typical high signal intensity in the affected disc on T2-weighted images and low signal on T1-weighted images, suggesting edema and inflammation (Figure 19) (119). Adjacent vertebral bodies may also appear hyperintense due to bone marrow edema, and contrast-enhanced MRI can further delineate the extent of infection or abscess formation (119, 120). In some cases, CT scans may show cortical bone destruction more clearly, though MRI remains more sensitive in detecting early changes. Blood tests, including inflammatory markers like C-reactive protein and erythrocyte sedimentation rate, are also helpful in supporting the diagnosis (121).

Treatment for discitis typically begins with antibiotics, which are chosen based on blood culture results when available and are often administered intravenously before transitioning to oral therapy. In cases where there is no clear infectious cause, anti-inflammatory medications may be used to manage symptoms. The duration of antibiotic treatment can vary but generally lasts from 4 to 6 weeks, depending on the severity of the infection and the patient's response to therapy (122). Surgical intervention may be necessary if there is an abscess, significant spinal instability, or neurological impairment (123). Physical therapy and other supportive measures are often recommended during recovery to improve function and reduce pain.

Recent advancements in imaging for discitis-osteomyelitis have introduced promising modalities that enhance diagnostic precision and patient management (124). The integration of 18F-fluorodeoxyglucose PET/CT leverages the metabolic imaging capabilities of PET/CT with the anatomical detail of MRI and offers improved sensitivity in detecting paravertebral and muscular abscesses, particularly in scenarios where MRI findings are inconclusive or when distinguishing between infectious and degenerative spinal conditions is challenging (125).

## 5 Vertebral lesions

### 5.1 Vertebral tumors

Vertebral tumors include both benign entities such as hemangioma, osteoid osteoma, and osteoblastoma as well as malignant lesions such as osseous metastases, osteosarcoma, chondrosarcoma, multiple myeloma, and lymphoma.

#### 5.1.1 Vertebral hemangioma

Hemangiomas are sporadic, benign neoplasms of blood vessels. Vertebral hemangiomas (VH) are the most common benign spinal axis tumors and are often detected incidentally on imaging. These tumors affect 10%–20% of adults with a 2:1 female preponderance and a higher prevalence after the 5th decade of life (126). Most VH are asymptomatic, but presenting symptoms may include localized back pain, numbness, and paresthesia. 20%–45% of symptomatic VH can display rapid bone expansion and destruction of surrounding bone and soft tissue beyond the vertebrae into the paravertebral and epidural space. This may lead to ischemia and vertebral body collapse (127). 45% of symptomatic cases may result in spinal cord and nerve root compression leading to neurologic deficits (127). VH may present with multi-level spine involvement at any spinal level, but symptomatic VH often present in the thoracic spine (127). 20%–30% of VH affect the thoracolumbar spine with extension into the vertebral body, pedicle, lamina or spinous process (127). For asymptomatic to mild lesions, management includes observation and pain control, while patients presenting with symptomatic lesions and neurologic deficits may require biopsy, surgery, radiotherapy, or preoperative embolization depending on radiologic findings and symptom severity (126).

Histologically, VH can be composed of blood vessels, adipocytes, smooth muscle, fibrous tissue, hemosiderin, interstitial edema, and remodeled bone (127) Microscopic characterization of VH includes capillary (50%), cavernous (28%), mixed (22%), arteriovenous, and venous subtypes (128). Capillary or cavernous VH subtypes are most common (127). Capillary hemangiomas arise from small, thin-walled blood vessels while cavernous hemangiomas arise from larger dilated vessels (126). Both subtypes are surrounded by edematous stroma and bony trabeculae within the marrow space (127). Bone remodeling can lead to thickened trabeculae of affected vertebrae which characteristically present as “polka-dot” sign on axial MRI/CT and “corduroy” sign on coronal & sagittal MRI/CT. VH may be commonly characterized on imaging with radiographs, CT, and MRI. Imaging groups include Typical, Atypical, and Aggressive subtypes (127).

**Figure 20:**
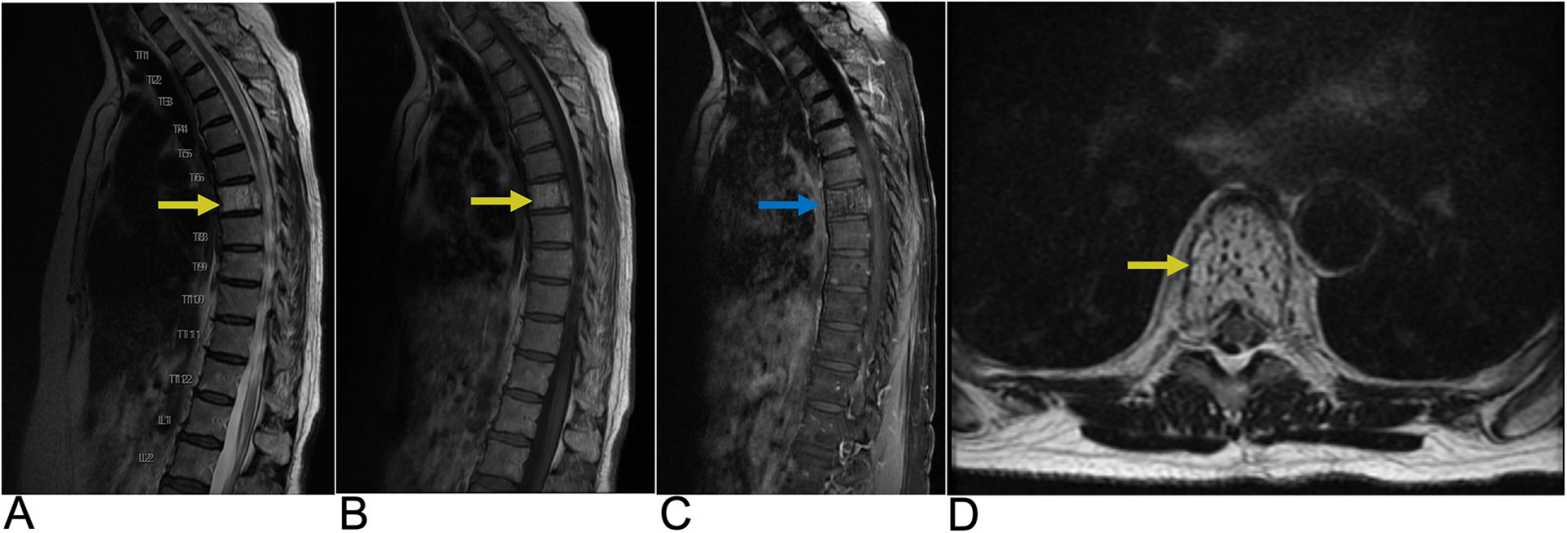
Vertebral hemangioma. Sagittal T2 **(A)**, sagittal T1 **(B)**, sagittal T1 FAT + C **(C)**, and axial T2 **(D)** MRI of the thoracic spine demonstrating a granular “salt and pepper” T1 & T2 hyperintense (yellow arrows), heterogeneously enhancing (blue arrow) lesion representing hemangioma of the T7 vertebral body.

Typical VH are easier to diagnose on imaging, display a low vascular content-to-fat ratio, and are generally asymptomatic, slow growing lesions that do not display extraosseous extension (127). Resorption of horizontal bone trabeculae and reinforcement of vertical trabeculae causes “corduroy cloth” appearance on radiographs, sagittal CT, and sagittal/coronal T1-WI & T2-WI. Small areas of thickened trabeculae surrounded by stroma may cause “polka-dot” or “salt and pepper” appearance of typical VH on unenhanced axial CT and axial T1-WI (Figure 20) (126, 127). Typical VH appear hyperintense on T1-WI & T2-WI as well as fluid sensitive MRI sequences. Typical VH are heterogeneously enhancing on T1-WI with contrast (127).

Atypical VH may not display classic “corduroy” or “polka-dot” signs due to having a higher vascular content-to-fat ratio. Atypical VH are also more likely to be aggressive and may mimic other spinal lesions such as multiple myeloma or metastatic lesions. Atypical VH are iso-hypointense on T1-WI and very hyperintense on T2-WI and fluid sensitive sequences (127).

Aggressive VH resembles atypical VH on imaging and may otherwise be radiographically normal or present with additional findings such as extension beyond the vertebral body, irregular trabeculae, cortex expansion, and bone destruction. CT findings may include involvement of the whole vertebral body or a honeycomb pattern due to vascular channels and fatty proliferation within remodeling bony trabeculae (127). Aggressive VH may be hypointense on T1-WI, hyperintense on T2-WI with possible intraosseous fatty stroma, and may be differentiated from metastases by high ADC values on DWI (127). Angiography may demonstrate vertebral body arteriole dilation, capillary phase blood pools, and complete vertebral body opacification characteristic of aggressive VH (127).

#### 5.1.2 Osteoid osteoma/osteoblastoma

Osteoid osteoma and osteoblastoma are similar benign primary osteogenic neoplasms accounting for 15% of benign skeletal neoplasms (129). Osteoid Osteoma (OO) often presents in patients under 5–25 years old years old with a 2:1 male predilection and comprises 10%–14% of primary vertebral tumors, 10%–12% of benign bone tumors, and 3% of primary bone tumors (129, 130). Spinal OO typically presents with nocturnal back pain and may present with scoliosis and radicular symptoms due to local inflammatory irritation of nerve roots. Regionally, spinal OO is most common in the lumbar region, then in the cervical and thoracic regions, followed by the sacrum. 75%–90% of spinal OO occurs on the posterior aspect of the spine. Spontaneous resolution of OOs may be seen with medical management alone. Other management options include surgery and CT guided percutaneous radiofrequency ablation (130). On CT a central, lucent nidus <1.5–2 cm of variable contrast enhancement is characteristic of OO. MRI may not display this nidus which can cause the OO to mimic other bone lesions (129, 130). This nidus is composed of osteoid islands filled with osteoblasts and is surrounded by a clear area of osteoclastic resorption and dense sclerotic bone (130).

**Figure 21:**
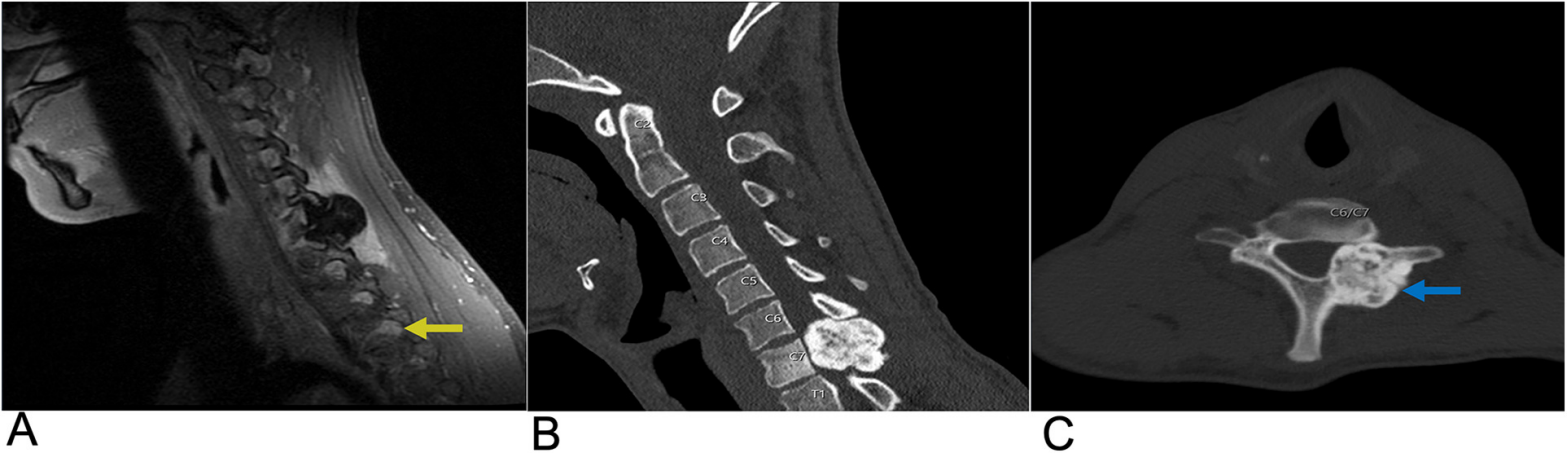
Osteoblastoma. Sagittal T1 + C MRI **(A)** as well as Sagittal CT **(B)** and Axial CT **(C)** of the cervical spine demonstrating a calcified enhancing lesion (yellow arrow) in the C7 left lateral mass representing osteoblastoma (blue arrow).

Osteoblastoma often presents in patients aged 10–25 years with a 2:1 male predilection and comprises 3% of benign bone tumors and 1% of all primary bone tumors. Osteoblastoma is typically larger than OO with a 4 cm diameter on average, do not spontaneously resolve, and more often become locally aggressive extending beyond the cortex (130). These lesions present with dull, aching progressive local pain and often involve posterior aspects of the spine with extraosseous extension leading to neurologic symptoms, scoliosis, or torticollis (129). Lumbar regions followed by thoracic, cervical, then sacral regions of the spine are most affected (130). Osteoblastoma require surgical management via intralesional curettage with or without cryoablation or chemical cauterization in most cases or enblock resection for larger tumors (129). Histologically, osteoblastoma have a disorganized central area surrounded by a less dense sclerotic reaction and a thin shell of less sclerotic periosteal bone relative to OO. More locally aggressive osteoblastoma display mitotically active large epithelioid osteoblasts on bony trabeculae and multifocal lesions (129). On CT, osteoblastoma is observed with a thin marginal bone shell and display intralesional mineralization centrally, cortical destruction, expansile bone remodeling, and reactive sclerosis peripherally (129). Although MRI may overestimate the extent of osteoblastoma due to local inflammation/edema, MRI remains useful to characterize spinal cord involvement and displays osteoblastoma as hypo-isointense on T1-WI and hyper-isointense on T2-WI (Figure 21) (129).

#### 5.1.3 Osteosarcoma

Osteosarcoma is the second most common primary bone tumor but occurs in the spine in only 0.85%–3% of cases which account for 3.6–14.5% of primary spinal tumors (131, 132). In white populations, osteosarcoma more commonly occurs in patients after the 6th decade, whereas in African American populations, the incidence is greater in patients aged 25–29 (132). Spinal osteosarcoma typically occurs in older age groups in the seventh decade with a male predilection (131, 132). Approximately 1% of patients with Paget's disease develop osteosarcoma and other risk factors include radiation therapy (132). Presenting symptoms include progressive pain disturbing sleep and neurologic deficiency in two-thirds of cases (132). The sacral region (30% of cases) followed by the lumber (25%) and thoracic (25%) spine are most commonly affected with 17% of cases involving >2 vertebral levels (131, 132). Additionally, osteosarcoma arises on posterior elements of the spine in 79% of cases (131). Spinal osteosarcoma may be managed via wide, enblock surgical resection with adjuvant, neoadjuvant, or primary local chemo- and radiation therapy with 5-year survival of 30%–40%, which is lower than osteosarcoma of other regions due to close proximity to spinal structures (132).

Histologically, osteosarcoma originates from osteoid producing mesodermal tissue and displays chondroblastic, osteoblastic (most common), small cell tumor, telangiectatic, and fibroblastic subtypes (132). Various somatic mutations and hereditary syndromes are associated with osteosarcoma including retinoblastoma, Li-Fraumeni syndrome, and Werner syndrome presenting potential therapeutic targets for novel chemotherapies (132).

**Figure 22:**
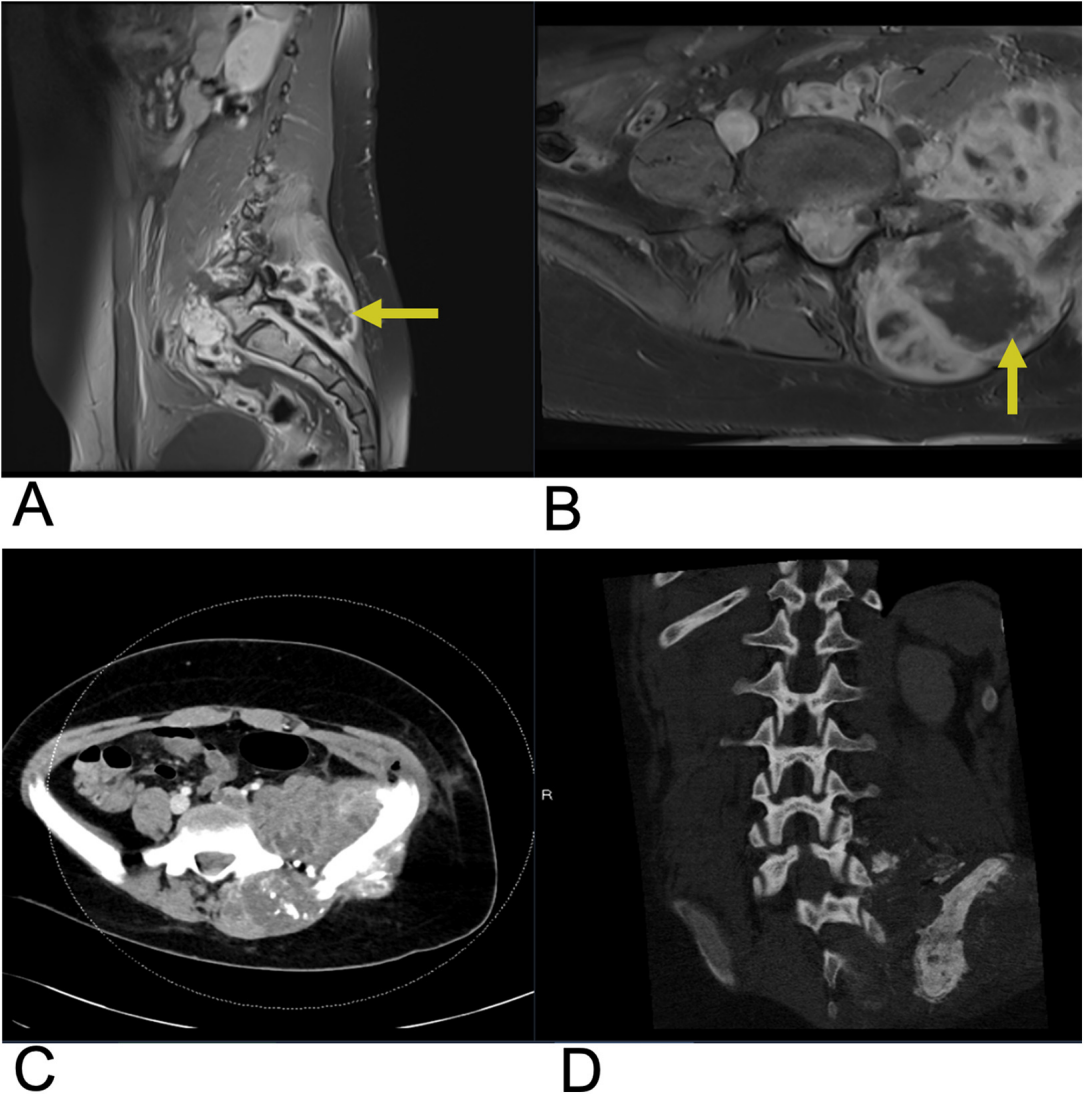
Osteosarcoma. Sagittal T1 + C **(A)**, Axial T1 + C **(B)** MRI as well as axial CT **(C)** and sagittal CT **(D)** of the lumbar spine demonstrating a large, destructive mass in the sacral region (yellow arrows) indicating osteosarcoma with adjacent soft tissue involvement.

On radiographs and CT, spinal osteosarcoma typically displays mixed osteoblastic/osteosclerotic and osteolytic lesions with components of variable ossification. Highly mineralized tumors may rarely present as “ivory vertebra”, while a purely lytic pattern may be seen in many subtypes including telangiectatic, mimicking aneurysmal bone cysts (131, 132). On MRI, spinal osteosarcoma may present with nonspecific intensities including hypointensity on T1 & T2-WI for mineralized lesions and hyperintensity on T2-WI for non-mineralized tumors (Figure 22) (131). Additional findings may include fluid-fluid levels in telangiectatic subtypes, cortical destruction, aggressive periosteal reaction (Sunburst, Codman Triangle), soft tissue extension, and pathological fracture (132). CT is the imaging modality of choice to characterize the mineralization pattern and cortical destruction of spinal osteosarcoma (132).

#### 5.1.4 Vertebral metastases

Spine metastases are found in 60%–70% of cases of systemic cancer via arterial, venous, CSF transmission, or direct extension (133). 80% of metastatic primary tumors spread to the spine from the breast (72%), prostate (84%), thyroid (50%), lung (31%), kidney (37%), and pancreas (33%) making the spine the third most common site of metastasis. Extradural spine metastases are most common (95% of cases) followed by intradural extramedullary (5%–6%), and intramedullary (0.5%–1%) locations (134). Approximately 10% of patients with spine metastases are symptomatic. Spine metastases may present with back pain followed by neurologic symptoms and cord compression from metastases to the vertebral column (85%), paravertebral region (10%–15%), and epidural or subarachnoid/intramedullary space (<5%) (134). Pain due to thick cortical bone damage is worse at night and responds to activity and low dose steroids, while pain due to acute pathologic fracture worsens with movement. Sensory/motor/autonomic neurologic symptoms vary with the sites of cord compression and/or nerve root compression. The most common sites for symptomatic cord compression include the thoracic (70%), lumbar (20%), and cervical (10%) regions (133). Management goals for spinal metastases are focused on improving health related quality of life and include pain control, preservation/restoration of neurologic function, and maintenance of spine stability. Treatment strategies include medical management, radiotherapy, and surgery with 2-year survival rates of 10%–20% from diagnosis (133).

Histopathological evaluation via image guided or open core biopsy is necessary to determine the best management strategy for spine metastases with inconclusive initial evaluation, while fine needle aspiration may be used if the primary tumor was previously identified (133). Radiosensitive tumors include hematological malignancies, round cell sarcoma, choriocarcinoma, small cell lung, and breast carcinoma, which may be treated with radiotherapy alone. Moderate radiosensitive tumors include colon and non-small cell carcinoma. Conversely, radioresistant tumors include sarcoma, renal carcinoma, and melanoma, which may require surgical decompression instead of radiotherapy (133).

Bone scintigraphy is the standard initial modality for imaging skeletal metastases. Most metastatic lesions have elevated radiotracer uptake (hot) indicating increased bone turnover, but some aggressive metastases may not uptake tracer (cold) due to poor blood flow or lack of reactive bone (134). Solitary foci of increased uptake indicate metastases in only 50% of cases, while multiple foci are strongly indicative of metastases. FDG PET/CT has a reported 98% sensitivity for the detection of spinal metastases and can differentiate osteoporotic from malignant vertebral compression fractures (134).

**Figure 23:**
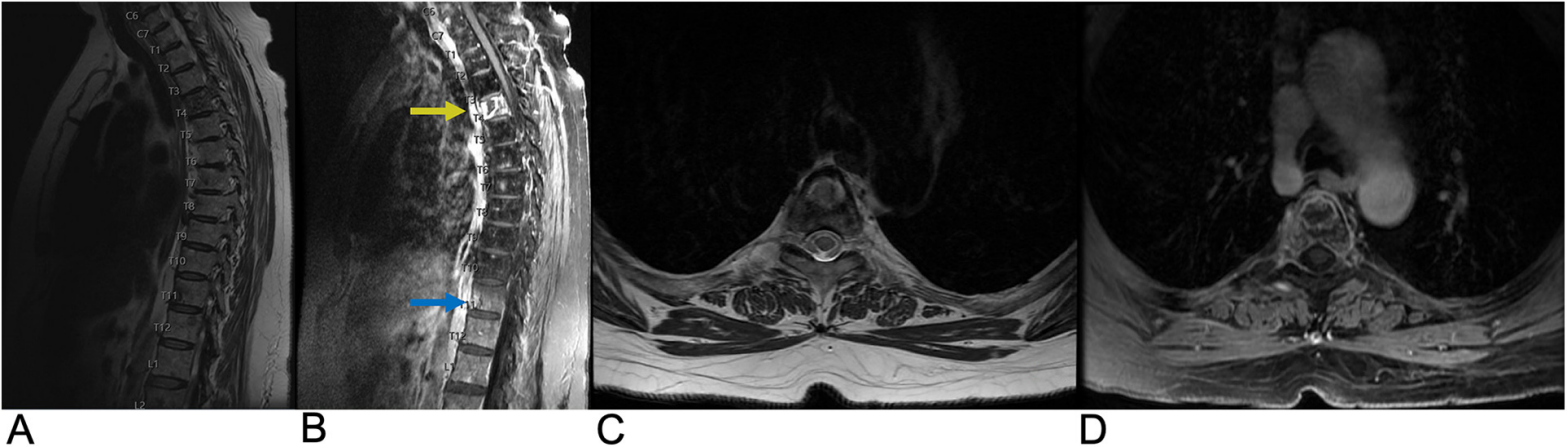
Vertebral metastases. Sagittal T2 **(A)**, sagittal T1 + C **(B)**, axial T2 **(C)**, and axial 3D FSPGR FAT + C **(D)** MRI showing destructive, enhancing lesions (yellow arrow) within the T4 vertebral body extending into the bilateral pedicles as well as an enhancing lesion (blue arrow) in the anterior aspect of the T11 vertebral body. There are also several smaller foci of enhancement scattered throughout the thoracic spine suspicious for smaller metastases.

CT is better suited to identify vertebral metastases and delineate cortical destruction than plain radiographs. However, lesions without bone destruction may be missed on CT. MRI was found to be more sensitive than CT for osseous metastases detection and better for delineating adjacent soft tissue and diagnosis in the setting of concurrent osteoporosis/degenerative changes (134). Additionally, T1-weighted spin echo and STIR sequences are used to identify hypointense malignant bone marrow abnormalities and may guide biopsy of these regions. On T2-WI, vertebral metastases are hyperintense relative to bone marrow with a rim of bright signal (halo sign) (134). Enhancing vertebral metastases on T1-WI with contrast indicate extradural, intradural extramedullary, or intramedullary lesions (Figure 23). T1-WI with contrast and fat suppression decreases background fatty signal from bone marrow resulting in increased intensity of metastatic lesions relative to bone marrow and nonneoplastic lesions (134).

#### 5.1.5 Multiple myeloma

Multiple myeloma (MM) is the third most common blood cancer in adults. The prevalence of MM is 4.3 cases per 100,000 individuals and is more common in males under the age of 30 with vertebral involvement in 65% of cases (131). MM is a neoplastic plasma cell disorder that develops from monoclonal gammopathy of unknown significance or smoldering myeloma. Any bone may be affected in MM necessitating a complete skeletal survey of the skull, spine, chest, pelvis, and appendicular skeleton. In cases with vertebral body involvement, extension into the pedicles is common and extension through the intervertebral disc is possible (131). Presenting symptoms include back pain, neurologic impairment, spinal cord compression resulting from extramedullary plasmacytoma or crush fracture secondary to lytic lesions, and rarely meningeal myelomatosis (135). Treatment modalities include systemic medical therapy as well as radiotherapy or surgery for focal lesions leading to neurologic compromise. Following treatment and resolution, T1-weighted MRI signal normalizes indicating an increased proportion of fatty marrow (135). Diagnosis of MM requires the following three clinical and histopathological criteria to be met: >10% monoclonal plasma cells in bone marrow or a biopsy proven plasmacytoma, monoclonal protein in the serum or urine, and one of the CRAB criteria (elevated serum calcium, renal insufficiency, anemia, & at least one lytic bone lesion (5 > mm) present on imaging) (135).

**Figure 24:**
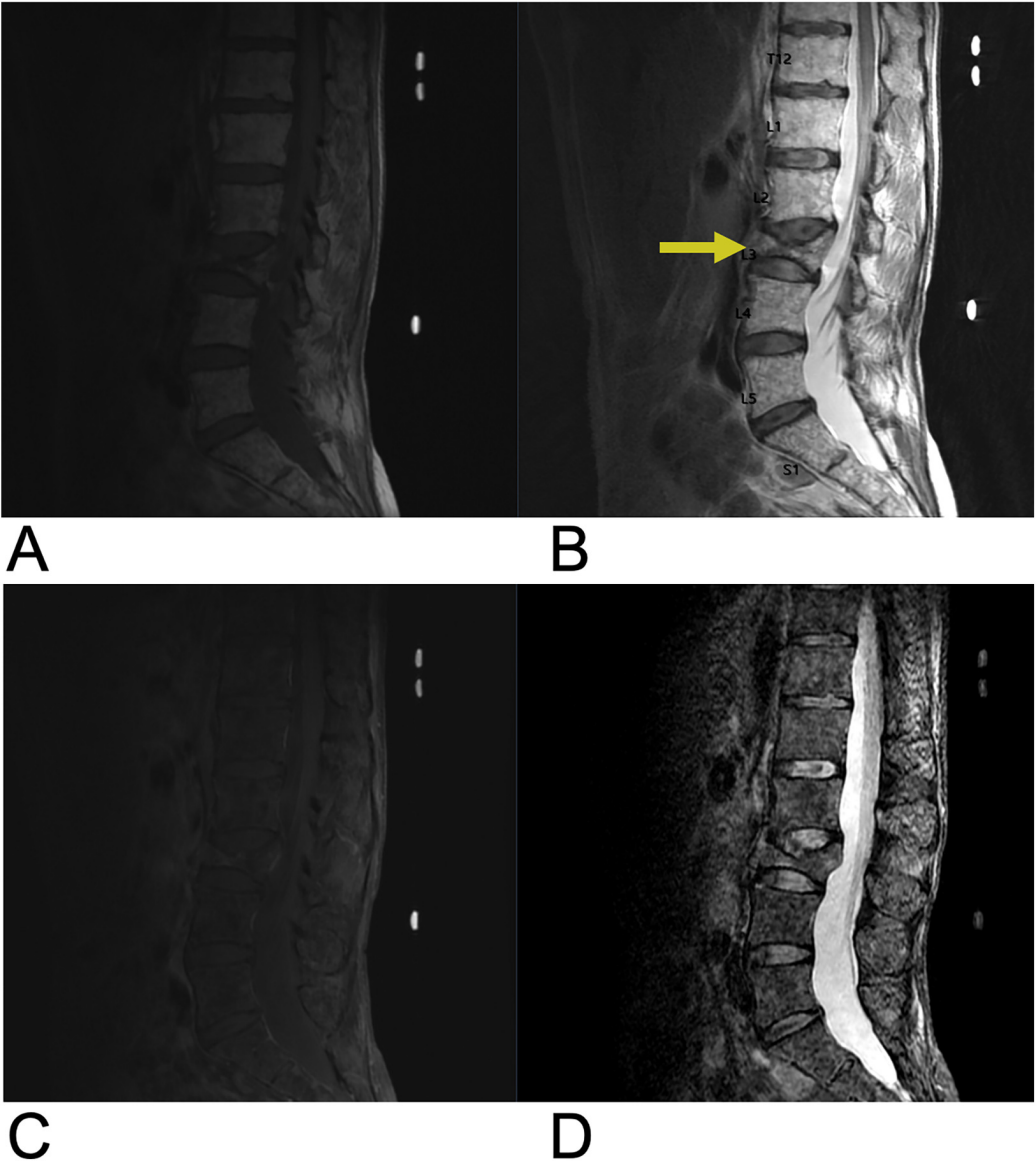
Multiple myeloma. Sagittal T1 **(A)**, sagittal T2 **(B)**, sagittal T1 + C **(C)**, and sagittal STIR **(D)** MRI of the lumbar spine demonstrating diffuse T1 hypointense, heterogenous T2 hyperintense, and T1 + C enhancing lesions representing vertebral manifestations of multiple myeloma. There is a chronic L3 superior endplate pathologic fracture with approximately 30% loss of vertebral body height (yellow arrow) and retropulsion of posterior vertebral body cortex.

Whole body x-ray (WBXR) has been the conventional imaging modality for MM. On WBXR, focal well-circumscribed lytic lesions, typically without reactive sclerosis or diffuse osteolysis, are seen. 10%–20% of MM may appear normal on radiographs (131). CT is preferred for fracture and compression fracture assessment due to greater sensitivity, but neither WBXR nor CT is useful for follow up of lytic lesions which remain unchanged upon remission (131, 135). A single lytic lesion on CT necessitates biopsy for MM diagnosis. Extramedullary plasmacytoma, which occurs in 13% of MM patients in soft tissues around the axial skeleton, may also be identified on CT (135). On MRI, MM lesions are typically hypointense on T1-WI or T2-WI, representing diffuse bone marrow infiltration, and hyperintense on T2-WI with fat suppression or T2 STIR, which unmasks focal lesions hidden by marrow infiltration (Figure 24) (135). MM lesions are contrast enhancing although contrast administration is not necessary for diagnosis. Five characteristic MR patterns of vertebral bone marrow in MM patients include normal and “salt and pepper” appearances, which indicate stage I disease, as well as diffuse infiltration, focal lesions, and combined focal/diffuse infiltration, which indicate higher stage disease (131, 135).

#### 5.1.6 Spinal lymphoma

Primary osseous lymphoma is a rare extra-nodal manifestation of non-Hodgkin lymphoma accounting for 1%–3% of lymphomas and 7% of primary bone malignancies (131). About 15% of osseous lymphoma present with spine involvement and 3% present with spinal cord compression. The age of diagnosis is 40–65 years (136). Primary osseous lymphoma is diagnosed if there is no other disease site for a minimum of six months (131). The vertebral body is often involved, and anterior elements are more commonly involved than posterior elements of the vertebral column (131, 136). Although contiguous vertebral involvement is possible, the disc space is usually preserved (131). Presenting symptoms may include back pain, weight loss, fever, fatigue, anorexia, cachexia, and constipation (136). Histopathological evaluation of lymphoma shows mixed cell infiltrate with heterogeneous cell size and shape as well as reactive sclerosis of bony trabeculae (131). Spinal lymphoma responds well to chemo- and radiation therapy and only requires surgery in cases presenting with neurologic deficits due to spinal cord compression and mechanical destabilization of the spine. Adjuvant radiotherapy is necessary if positive surgical margins remain, and adjuvant chemotherapy may also be administered.

**Figure 25:**
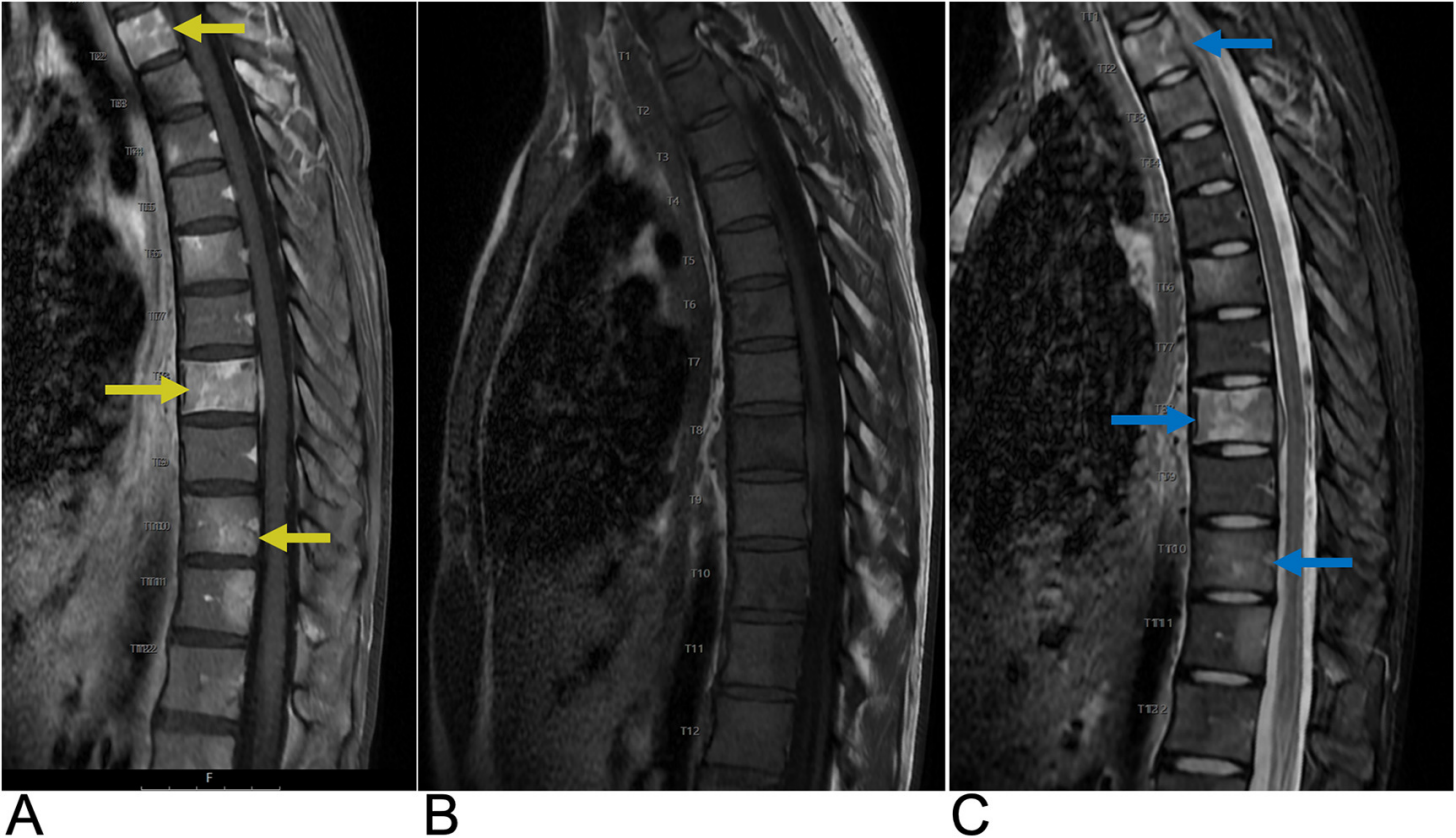
Spinal lymphoma. Sagittal T1 + C **(A)**, sagittal T2 **(B)**, and sagittal STIR **(C)** MRI demonstrating heterogeneous, intermediate T2 signal intensity changes, contrast enhancement (yellow arrows), and STIR hyperintensity (blue arrow) in the involved vertebrae indicative of lymphomatous infiltration.

On CT, bone lesions resulting from osseous lymphoma may be commonly lytic, mixed, or rarely sclerotic, and cortical thickening with periosteal reaction or pathologic fracture may also be seen. On MRI, osseous lymphoma presents with hypointensity on T1-WI, hyperintensity on T2-WI with fat suppression, and variable post contrast enhancement (Figure 25) (131). Additionally, FDG PET/CT may show focal or multifocal radiotracer uptake with a non-focal/diffuse background. A completely non-focal/diffuse background may indicate post-treatment hematopoietic response (131).

### 5.2 Vertebral compression fracture

Vertebral fractures are the earliest and most common fractures seen in patients with osteoporosis with increasing prevalence in individuals over 50 years of age (137). According to the European prospective osteoporosis study (EPOS), the vertebral fracture rate for women aged 50–79 (1.1% per year) was 1.83 times greater than the rate for men of the same age group (0.6% per year) (138). A prior vertebral fracture confers 5–12.6 times risk for future vertebral fractures and 2.3–3.4 times risk for future hip fractures (137). Presenting symptoms for vertebral fractures include back pain, limited movement, and physical deformity leading to decline in social function, impaired quality of life, and increased morbidity & mortality. However, 34% of vertebral fractures remain underdiagnosed due to nonspecific presentation, and of radiologically diagnosed cases, only 25% received further treatment for osteoporosis (137).

**Figure 26:**
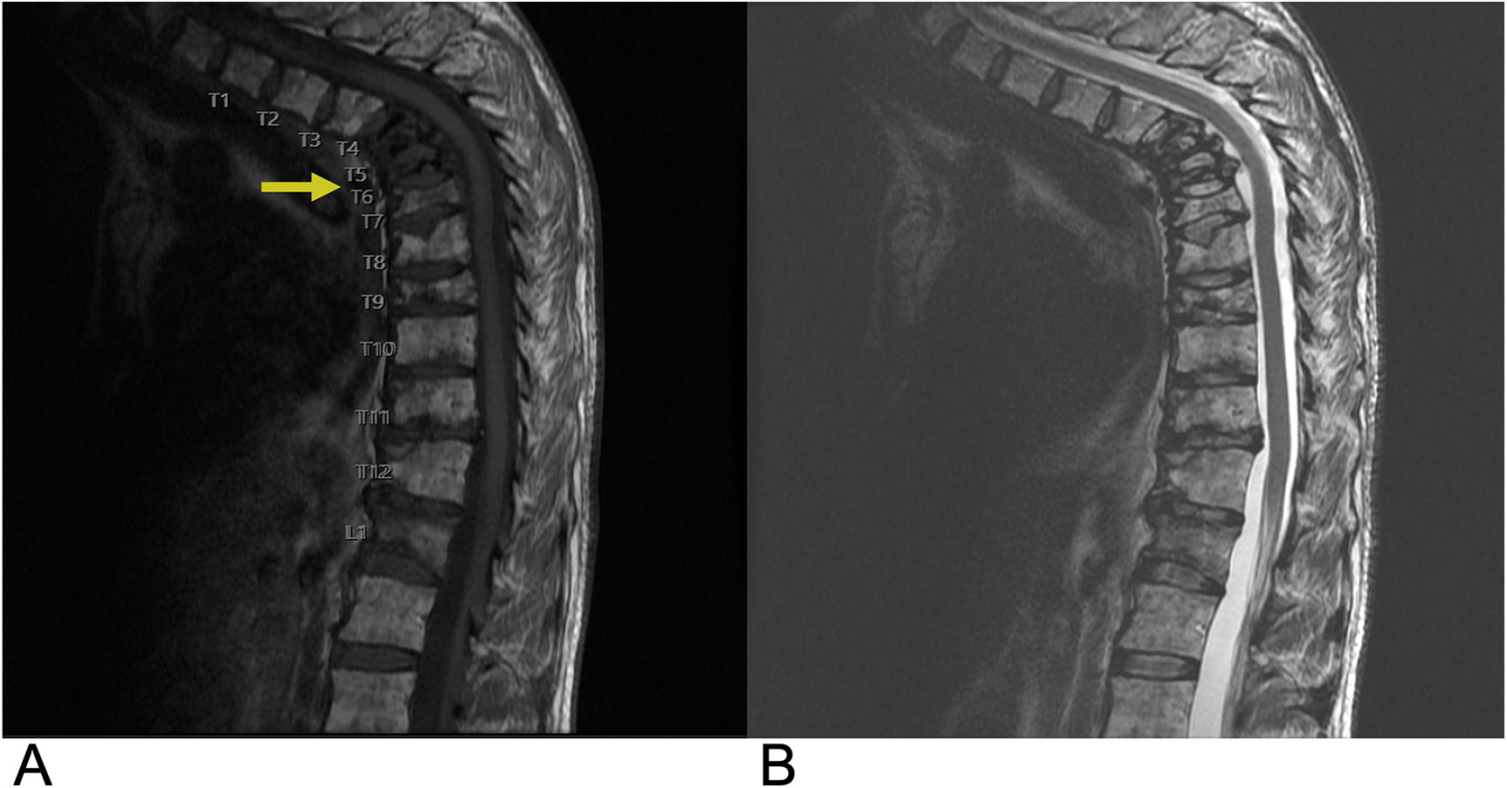
Vertebral compression fractures. Sagittal T1 **(A)** and sagittal STIR **(B)** MRI of the thoracic spine demonstrating osteoporotic compression fracture of upper thoracic vertebrae (yellow arrow) with height loss.

Vertebral fragility fractures in the setting of osteoporosis may occur secondary to an axial/compressive load. The mid-thoracic (T7–T8) and thoracolumbar (T12–L1) regions are most often affected by vertebral compression fractures. Plain radiographs are often sufficient for diagnosis. Improper oblique positioning of the spine on lateral thoracic (T7) and lumbar (L3) radiographs can lead to misinterpretation of scoliosis or obliquely positioned vertebral endplates as fracture/deformity (“bean-can effect”) (137). On correctly positioned radiographs, vertebral fractures can be identified by the presence of end plate deformities, altered/non-uniform vertebral size/shape, or loss of vertebral height >20% (137). Additionally, the Genant visual semi-quantitative method is an easily implementable means to identify moderate to severe fractures. This method involves visual assessment of vertebral height reduction due to wedge, biconcave, or crush deformities on radiographs to grade fractures on a 1–3 scale. Grade 1 fractures are classified as mildly deformed and display 20%–25% height reduction. Grade 2 fractures are classified as moderately deformed and display 25%–40% height reduction. Grade 3 fractures are classified as severely deformed and display >40% height reduction (137). This method omits specific deformity as a criterion for grading but still accounts for the impact of overall morphologic changes via height reduction as a proxy measure (Figure 26). A “spinal fracture index” as the average grade for all vertebra evaluated can be calculated for prognostic purposes (137).

## 6 Prevertebral space

Prevertebral space spinal lesions are rare and can arise from a variety of etiologies, including infectious, neoplastic, and inflammatory conditions (139). Common lesions in this space include neoplasms such as metastases from head and neck cancers, thyroid carcinoma, or lymphoma, and abscesses typically secondary to vertebral osteomyelitis or discitis. Imaging with MRI is essential for diagnosis, often showing a mass in the prevertebral space with potential spinal extension or compression, and T2-weighted hyperintensity for both abscesses and tumors (140). Early diagnosis and management are crucial to prevent severe complications such as airway obstruction or irreversible spinal cord damage.

### 6.1 Prevertebral space neoplasm

Prevertebral space (PVS) neoplasms can arise from both primary and secondary sources, with secondary metastases being more common. Metastatic lesions often originate from primary lung or breast cancers and can extend into the prevertebral space, causing mass effect and potential spinal cord compression (141). These lesions typically exhibit T1 hypointensity, T2 hyperintensity, and contrast enhancement on T1-weighted images. Primary neoplasms with osseous involvement in the PVS include hematologic malignancies such as leukemia, lymphoma, and multiple myeloma, which commonly affect the bones in this region (7). Lipoma is the most common primary benign neoplasm of the PVS, characterized by its hyperintense T1 signal that saturates with fat suppression techniques on MRI. Malignant primary neoplasms, such as liposarcoma, are exceedingly rare in this space but can be more aggressive and require prompt diagnosis and management (142). Imaging is crucial in differentiating these lesions based on their specific MRI signal characteristics, which guide further diagnostic and therapeutic interventions. Neoplastic lesions can manifest with symptoms of spinal cord compression, radicular pain, or dysphagia (143), and are managed with a combination of surgical resection, radiation, and/or chemotherapy depending on the tumor type (144).

In recent years, the implementation of a combined PET/MRI course of imaging allows for more precise delineation of tumor boundaries and assessment of infiltration into adjacent structures, which is particularly beneficial in complex anatomical regions like the PVS.

### 6.2 Retropharyngeal abscess

**Figure 27:**
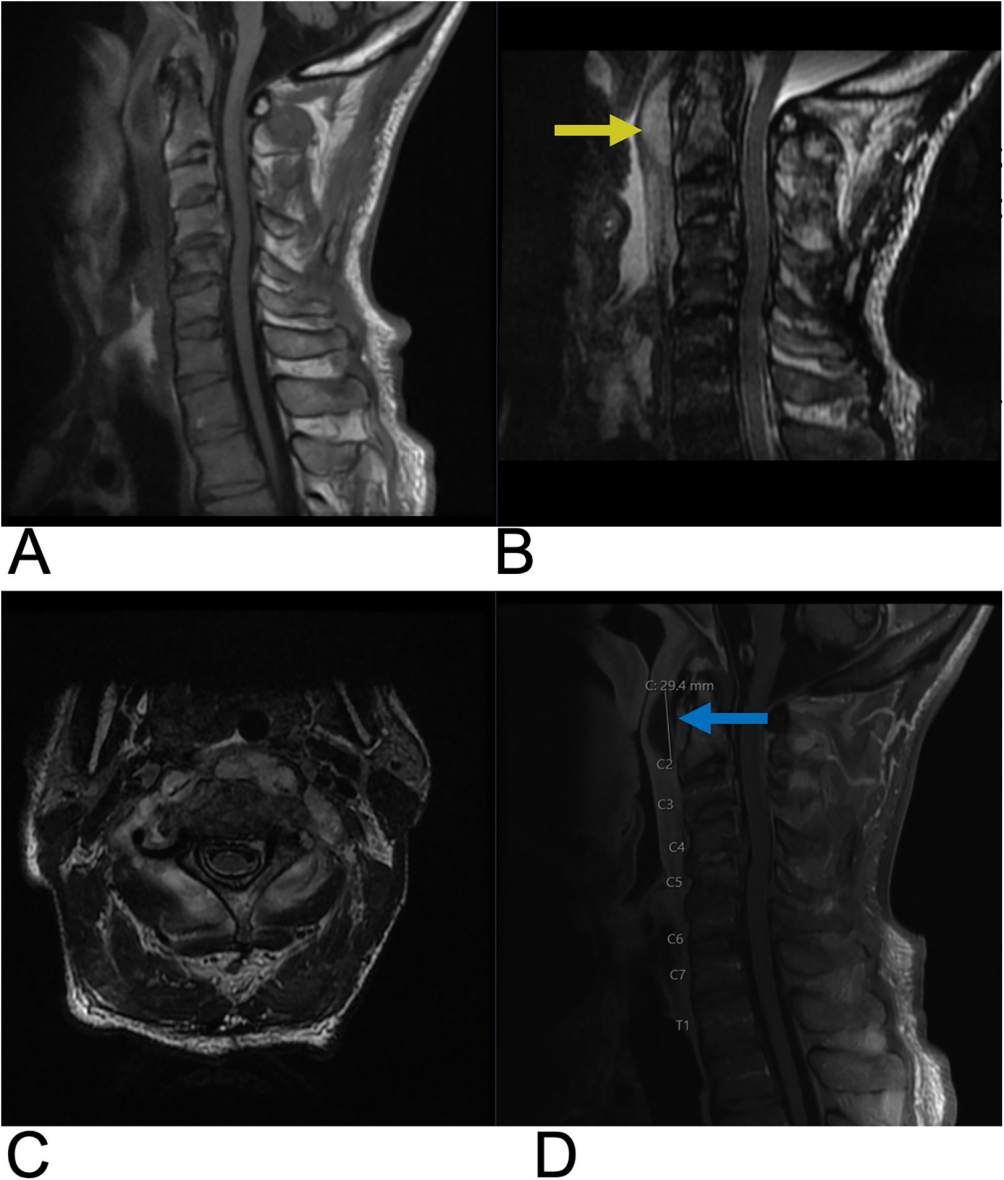
Retropharyngeal abscess. Sagittal T1 **(A)**, sagittal T2 **(B)**, axial T1 **(C)**, and sagittal T1 + C **(D)** MRI of the cervical spine demonstrating a retropharyngeal abscess appearing as a T2 hyperintense fluid collection (yellow arrow), with peripheral enhancement (blue arrow) located posterior to the pharynx and anterior to the cervical spine with significant prevertebral soft tissue swelling causing displacement of the spinal cord at the C3–C4 level.

Retropharyngeal space abscesses, while uncommon, represent a potentially life-threatening condition that demands prompt recognition and treatment. The most common underlying mechanism for these abscesses is infection, often originating from adjacent structures such as the vertebrae or oropharynx (145). Infectious lesions like prevertebral abscesses often present with fever, neck pain, dysphagia, and, in severe cases, airway compromise (146). Contrast-enhanced MRI reveals rim enhancement, distinguishing these from solid neoplastic lesions. A plain radiograph may reveal increased prevertebral space thickness, raising suspicion for an underlying abscess, but computed tomography (CT) is the preferred imaging modality due to its rapid turnaround time and superior spatial resolution (147). On MRI, these abscesses typically exhibit low T1 signal intensity and high T2 signal, helping to differentiate them from other prevertebral lesions (Figure 27). Treatment of prevertebral lesions depends on the underlying cause. Infections often require surgical drainage and antibiotic therapy. Early diagnosis and management, usually involving surgical drainage and antibiotic therapy, are critical to prevent complications such as airway obstruction, mediastinal spread, or spinal cord compression (7).

## 7 Incidental lesions

Incidental spinal lesions are asymptomatic abnormalities discovered during imaging for unrelated conditions and are increasingly identified due to the widespread use of advanced imaging techniques like MRI and CT (148). These lesions can encompass a wide range of pathologies, from benign findings such as cholelithiasis and hemangiomas, which are the most common benign vertebral neoplasms, to more concerning lesions such as small metastases or primary tumors. Imaging characteristics on MRI, such as high T1-weighted signal in hemangiomas or low T1-weighted signal in bone metastases, help in differentiating these lesions (149). Close radiological follow-up may be advised for indeterminate lesions or those with potentially malignant features, such as irregular borders or abnormal contrast enhancement (150). Notably, specific cancers can also be identified incidentally, with kidney, pancreatic, and rectal cancers often found when imaging the thoracic and lumbar spine as well as thyroid cancer frequently identified from imaging of the cervical spine (151, 152).

### 7.1 Congenital vertebral lesions

**Figure 28:**
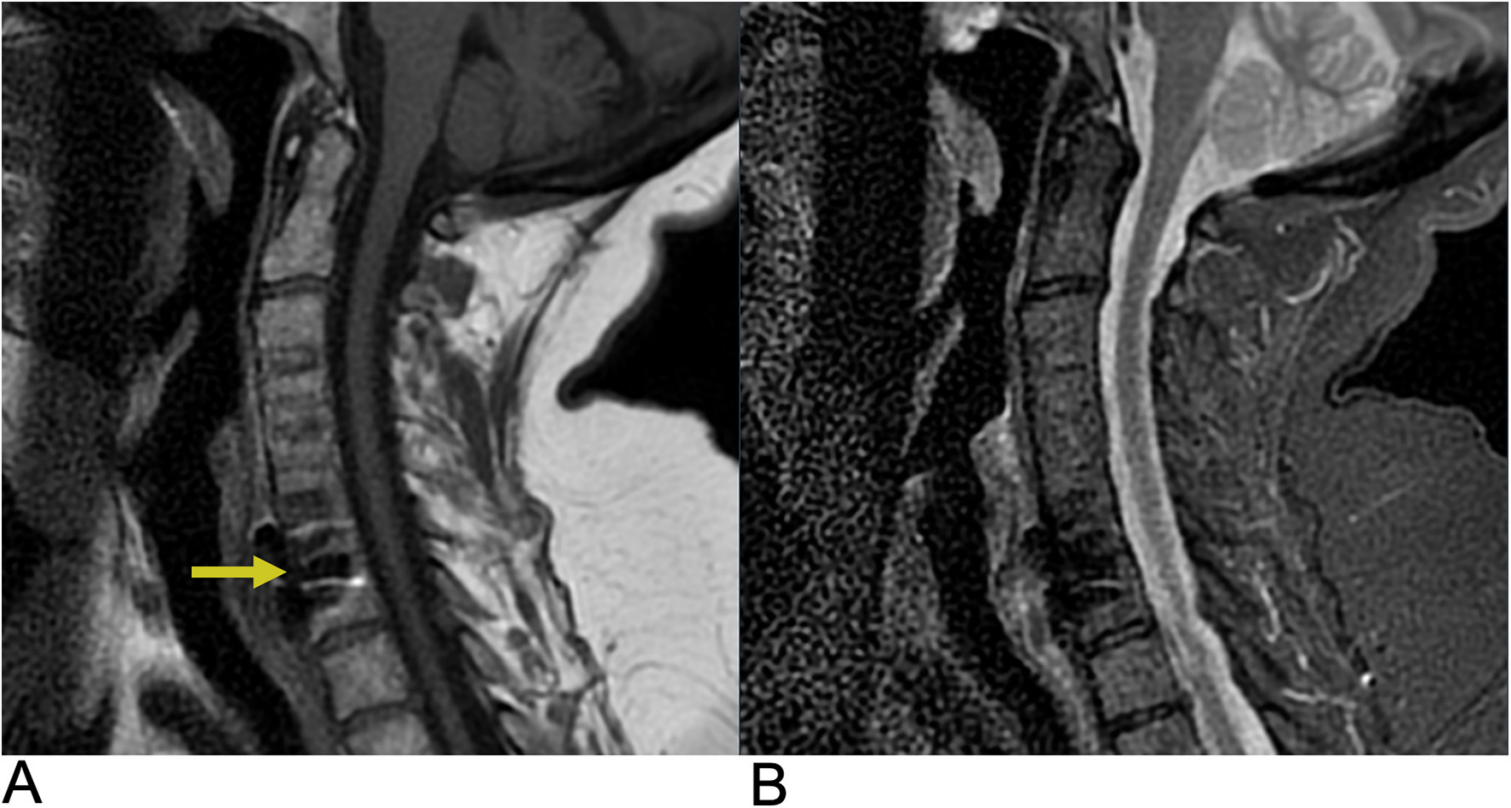
Block vertebra. Sagittal T1 **(A)** and sagittal T2 **(B)** MRI demonstrate absent intervertebral disc space due to congenital block vertebra resulting from failed vertebral segmentation. There is T1 hypointensity (yellow arrow) and T2 hyperintensity in adjacent soft tissues suggesting secondary changes or abnormal spinal curvature.

**Figure 29:**
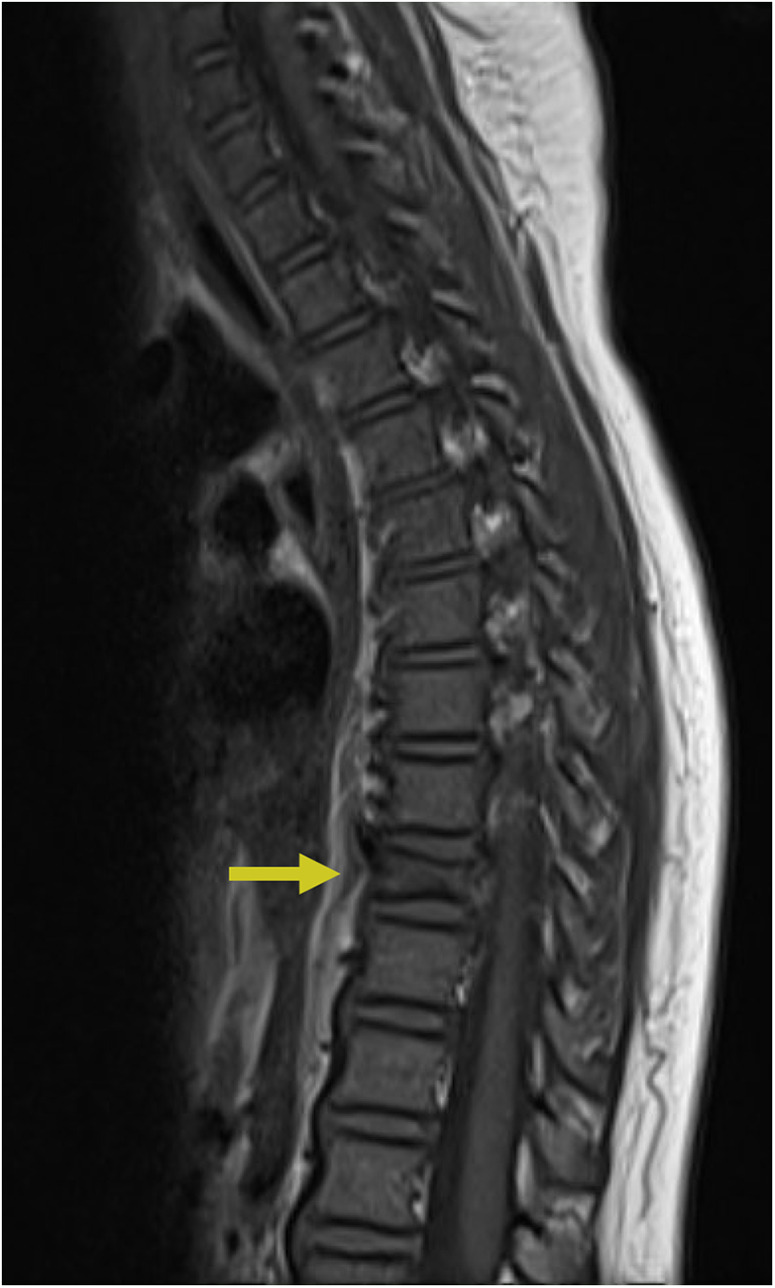
Congenital butterfly vertebra. Sagittal T2 MRI of the lumbar spine demonstrating a congenital T9 thoracic butterfly vertebra.

Block vertebrae are congenital spinal anomalies resulting from the failure of vertebral segmentation during embryonic development (153). Though often asymptomatic, block vertebrae can cause restricted spinal mobility or contribute to degenerative changes in adjacent spinal segments. On MRI, block vertebrae are characterized by the absence of the normal intervertebral disc and a continuous fusion of the vertebral bodies (Figure 28), which may involve the posterior elements as well. Chondrification or ossification failure is another congenital vertebral defect where parts of the vertebrae fail to form, leading to abnormal vertebral anatomy. The most notable manifestation of this failure is the presence of hemivertebrae, in which only one half of the vertebral body develops (154). In rare cases of failed sagittal fusion of the lateral halves of a vertebral body during embryonic development, two hemivertebra arise at the same vertebral level resulting in a characteristic “butterfly vertebra” pattern on imaging (Figure 29) (155). These defects can lead to back pain and significant spinal deformities, such as kyphosis or scoliosis, due to the asymmetrical growth of the spine.

Progressive improvements in resolution capacity of MRI and CT are particularly salient in bettering the identification of anomalies such as hemivertebrae, block vertebrae, and butterfly vertebrae (156). Advancements in prenatal imaging have additionally improved the early detection of such congenital vertebral malformations through real-time ultrasonography and fusion imaging complemented by fetal MRI, enabling identification *in utero* (157).

### 7.2 Miscellaneous (osteoporosis, degenerative endplate changes, enostosis (“Bone Islands”)

**Figure 30:**
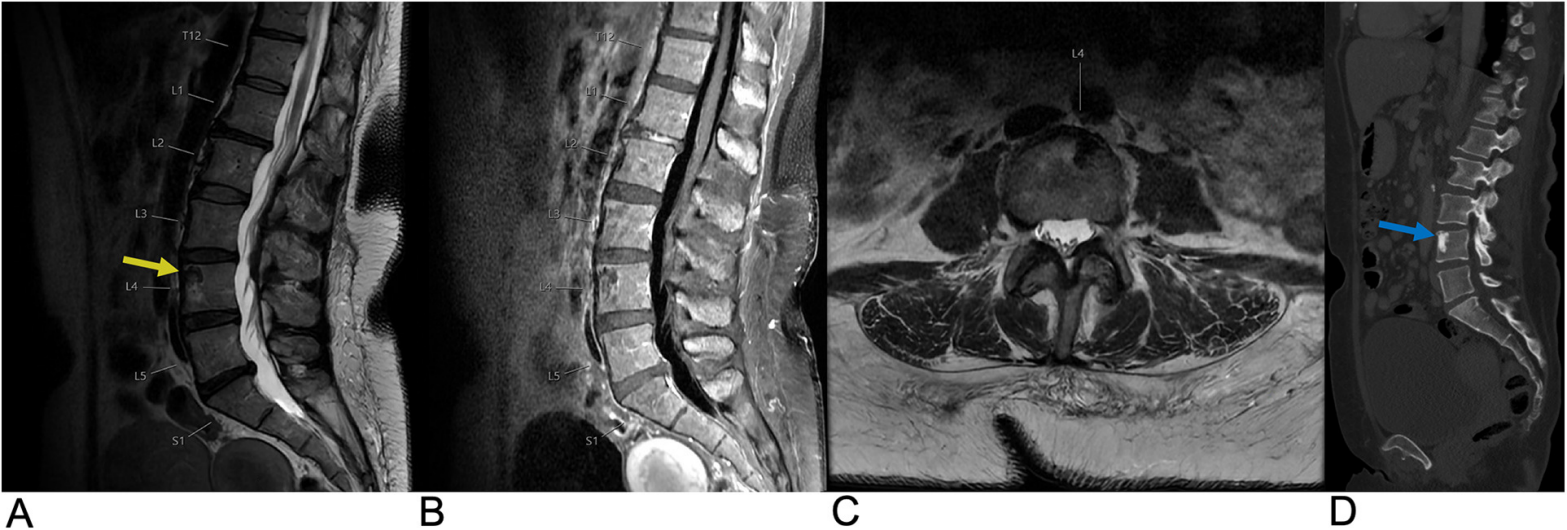
Enostosis. Sagittal T2 **(A)**, sagittal T1 FLAIR FAT + C **(B)**, and axial T2 **(C)** MRI as well as a sagittal CT **(D)** of the lumbar spine demonstrating a T1/T2 hypointense, sclerotic (yellow arrow) and CT hyperdense (blue arrow) lesion within the left anterior aspect of the L4 vertebral body representing enostosis.

Findings not classified above can range from indications such as osteoporosis, degenerative endplate changes, and bone islands. Osteoporosis is one of the most common conditions affecting the spine, particularly in older adults, and often remains asymptomatic until complications such as vertebral compression fractures occur (158). On MRI, osteoporosis is characterized by increased fat content in the bone marrow, leading to hyperintense signals on T1-weighted images (159). This is due to the replacement of normal hematopoietic marrow with fatty marrow as a consequence of the bone loss. Degenerative endplate (Modic) changes are a common finding in patients with chronic back pain and represent different stages of vertebral endplate degeneration (160). These changes are classified into three types based on MRI signal characteristics. Type 1 is characterized by T1 hypointensity and T2 hyperintensity, reflecting bone marrow edema and inflammation. Type 2 changes involve fatty infiltration of the bone marrow, presenting as hyperintensity on both T1 and T2-weighted images. Type 3 is the most advanced stage, showing T1 and T2 signal hypointensity due to sclerosis of the vertebral endplate (161). Enostosis, commonly referred to as a “bone island”, is a benign focus of compact cortical bone located within cancellous bone (162). These lesions are typically asymptomatic and discovered incidentally during imaging for other conditions (163). On MRI, enostosis exhibits signal characteristics similar to cortical bone, with low signal intensity on both T1 and T2-weighted images due to the dense, mineralized nature of the lesion (Figure 30) (164).

## 8 Conclusion

The focus of this article is to clearly present a range of spinal lesions, with an emphasis on their imaging characteristics and the specific spinal regions they affect. Diagnostic imaging plays a crucial role in evaluating the extent of spinal lesions, assessing their impact on adjacent neural structures, and identifying potential metastasis to other regions such as the lungs, liver, bone, and brain. Imaging characteristics along with histopathological data and information on metastatic spread, are essential for accurate staging, prognosis, and treatment planning of spinal lesions. This literature review provides an in-depth exploration of various spinal lesions, offering a comprehensive reference for practicing radiologists to review the imaging findings, anatomical implications, and clinical relevance of these lesions.
